# Atomistic Probing of Defect-Engineered 2H-MoTe_2_ Monolayers

**DOI:** 10.1021/acsnano.3c08606

**Published:** 2024-02-20

**Authors:** Odongo Francis Ngome Okello, Dong-Hwan Yang, Seung-Young Seo, Jewook Park, Gunho Moon, Dongwon Shin, Yu-Seong Chu, Sejung Yang, Teruyasu Mizoguchi, Moon-Ho Jo, Si-Young Choi

**Affiliations:** † Department of Materials Science and Engineering, Pohang University of Science and Technology (POSTECH), 77 Cheongam-ro, Nam-gu, Pohang-si 37673, Republic of Korea; ‡ Samsung Electronics, Foundry Analysis & Engineering Team, Global Manufacturing & Infra Technology, Samsungjeonja-ro 1, Hwaseong-si 18448, Republic of Korea; § Center for Van der Waals Quantum Solids, Institute of Basic Science (IBS), 77 Cheongam-ro, Nam-gu, Pohang-si 37673, Republic of Korea; ∥ Materials Science and Technology Division, Oak Ridge National Laboratory (ORNL), Oak Ridge, Tennessee 37831, United States; ⊥ Division of Biomedical Engineering, College of Health Sciences, Yonsei University, 1, Yeonsedae-gil, Heungeop-myeon, Wonju-si 26493, Republic of Korea; # Department of Precision Medicine, Yonsei University, Wonju College of Medicine, 20 Ilsan-ro, Wonju-si 26426, Republic of Korea; ∇ Department of Medical Informatics and Biostatistics, Graduate School, Yonsei University, 20 Ilsan-ro, Wonju-si 26426, Republic of Korea; ■ Institute of Industrial Science, The University of Tokyo, Komaba, Meguro 4-6-1, Tokyo 153-8505, Japan; ○ Department of Semiconductor Engineering, POSTECH, 77 Cheongam-ro, Nam-gu, Pohang-si 37673, Republic of Korea

**Keywords:** 2H-MoTe_2_, point defect, vacuum-annealing, laser-illumination, scanning transmission electron microscopy, deep learning

## Abstract

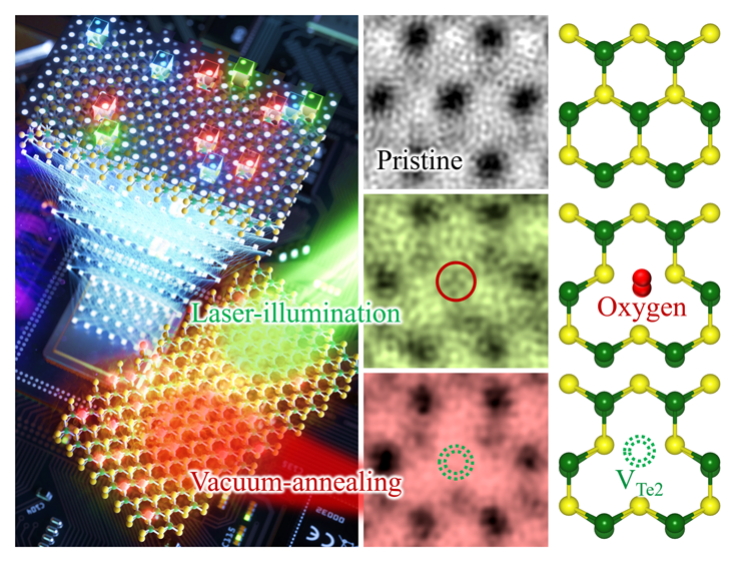

Point defects dictate various physical, chemical, and optoelectronic properties of two-dimensional (2D) materials, and therefore, a rudimentary understanding of the formation and spatial distribution of point defects is a key to advancement in 2D material-based nanotechnology. In this work, we performed the demonstration to directly probe the point defects in 2H-MoTe_2_ monolayers that are tactically exposed to (i) 200 °C-vacuum-annealing and (ii) 532 nm-laser-illumination; and accordingly, we utilize a deep learning algorithm to classify and quantify the generated point defects. We discovered that tellurium-related defects are mainly generated in both 2H-MoTe_2_ samples; but interestingly, 200 °C-vacuum-annealing and 532 nm-laser-illumination modulate a strong n-type and strong p-type 2H-MoTe_2,_ respectively. While 200 °C-vacuum-annealing generates tellurium vacancies or tellurium adatoms, 532 nm-laser-illumination prompts oxygen atoms to be adsorbed/chemisorbed at tellurium vacancies, giving rise to the p-type characteristic. This work significantly advances the current understanding of point defect engineering in 2H-MoTe_2_ monolayers and other 2D materials, which is critical for developing nanoscale devices with desired functionality.

## Introduction

As the channels in modern electronic devices become increasingly ultrathin with a target gate-length of less than 5 nm, the application of conventional Si-based transistors encounters certain bottlenecks, *e.g.*, the carrier mobility is compromised by surface-roughness-induced scattering and impeded by quantum-mechanical source–drain tunneling effect.^1−3^ The discovery of atomically thin two-dimensional (2D) materials such as trigonal-prismatic-coordinated hexagonal molybdenum ditelluride (2H-MoTe_2_) with distinctive tunable electric and optoelectronic properties presents an alternative solution to Si-based electronic devices.^4,5^ Moreover, thickness controllability in 2H-MoTe_2_ is ideal for highly scalable field-effect transistors (FETs) with significantly reduced short-channel effects while ensuring a high carrier mobility critical for exceptional performance at low-voltage device operations.^6−8^ Recently, several studies have suggested that the optoelectronic properties of 2H-MoTe_2_ and other 2D materials can be enhanced through meticulous control of atomic defects.^9,10^ This possibility has been widely demonstrated by employing strategic engineering techniques, *e.g.*, laser-illumination,^11^ vacuum-annealing, and chemical functionalization by oxygen.^12^ Subsequent structural and electrical investigations indicate that such engineering technologies can manifest 2H-MoTe_2_ with either n- or p-type electrical properties by generating a specific type of extrinsic structural defects.^13^

Despite such advancements in defect-engineering techniques, a comprehensive understanding of the correlation between defect structure–property modulation in 2H-MoTe_2_ and other 2D materials remains insufficient. Atomically thin 2D materials are prone to structural degradation, which often result in a compromised optoelectronic property;^14−16^ therefore, a continuous search for nondestructive and efficient defect-engineering technologies is still ongoing. Fifth-order spherical aberration-corrected scanning transmission electron microscopy (5^th^-order *C*_*s*_-corrected STEM) has been an indispensable tool to enable the visualization and study of defect dynamics with high-speed subpicometer resolution.^17,18^ However, the large amount of data generated during atomic structural imaging and the complexity of the structural defects make it tortuous, time-consuming, and nearly impossible to identify defect species with a high precision.^19,20^ Therefore, there is a compelling need to combine automated defect identification and classification algorithm, *e.g.*, deep learning, to study structure–property interdependence in defect-engineered 2D materials.^21,22^

Herein, we exposed 2H-MoTe_2_ monolayers (MLs) to different treatment conditions: 200 °C-vacuum-annealing and 532 nm-laser-illumination. The structural modification conditions were judiciously monitored to avoid severe structural degradation or phase transformation. Thereafter, we employed a synergistic combination of (i) a low-voltage 5^th^-order *C*_*s*_-corrected STEM and (ii) a deep learning-based analytic platform to systematically identify and quantify structural defects in the defect-modulated 2H-MoTe_2_ MLs. To elucidate the impact of atomic defects on the electrical property-modulation of 2H-MoTe_2_, we employed scanning tunneling spectroscopy (STS) to conduct electrical measurements by probing the variation in the local density of states (LDOS) near the defective sites in each defect-engineered 2H-MoTe_2_, exposed to 200 °C-vacuum-annealing and 532 nm-laser-illumination. Our results demonstrate that 2H-MoTe_2_ exposed to 200 °C-vacuum-annealing exhibits the strong n-type character, while 532 nm-laser-illuminated 2H-MoTe_2_ exhibits the strong p-type character. The direct investigation of each defect-engineering technology, as well as the impact of such generated defect species on the electronic properties of 2H-MoTe_2_, will act as a roadmap for designing high-performance structure-engineered 2D material-based back-gated FET transistor logic.

## Results and Discussion

**Figure 1:**
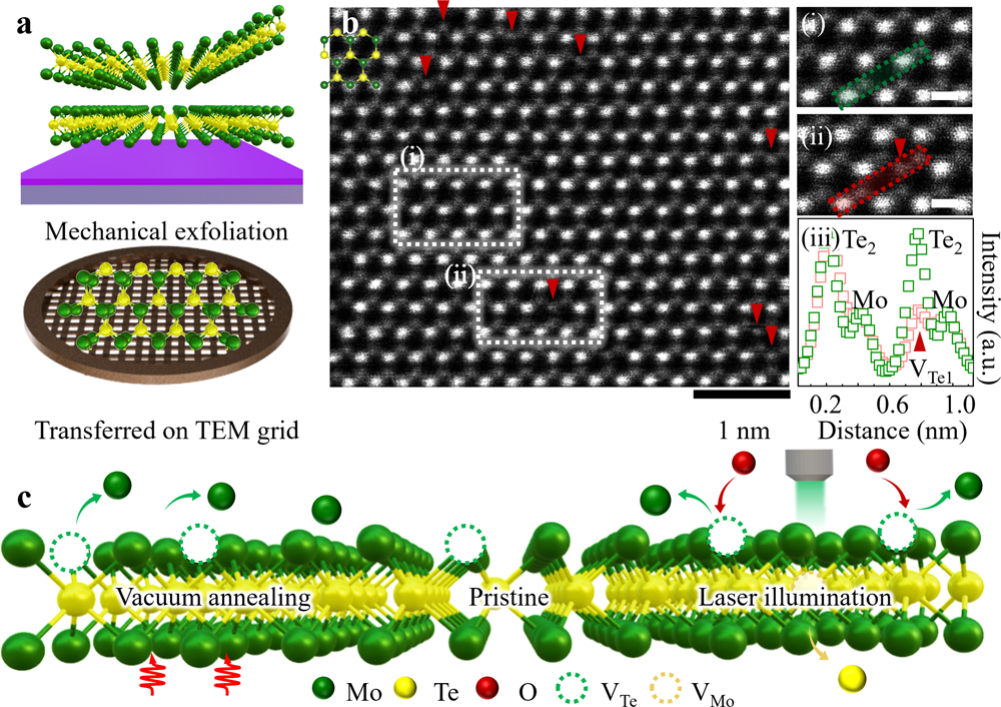
Strategy for defect-engineering. (a) Schematic illustration of the 2H-MoTe_2_ ML sample prepared by mechanical exfoliation (top) and then transferred onto a TEM grid (bottom). (b) Wide-view HAADF-STEM image of the pristine 2H-MoTe_2_ ML. The bright (dark) contrast regions denote Te_2_- (Mo) atomic columns. (i, ii) Enlarged HAADF-STEM micrographs in (b), revealing perfect and defective (V_Te1_/red arrow) lattices, respectively. Scale bars: 0.2 nm. (iii) Intensity profiles for Perfect (open green squares) and V_Te1_ (open red squares) regions extracted along the color-dotted diagonal rectangles in (i) and (ii), respectively. (c) Defect modulation of 2H-MoTe_2_ MLs; (left) vacuum-annealing, (middle) pristine, and (right) laser-illumination. Mo, green sphere; Te, yellow sphere; O, red sphere; V_Te_, green dotted circle; and V_Mo_, and yellow dotted circle.

To investigate point defects in 2H-MoTe_2_ ML, clean and high-crystalline samples are a prerequisite. Therefore, we adopted a mechanical exfoliation method for sample preparation and verified the crystallinity of the samples using a 5^th^-order *C*_*s*_-corrected STEM operated at 80 kV. Based on clear and high-resolution STEM images, it is possible to achieve (i) feasible defect-engineering and intuitive defect identification and (ii) deep learning-based point defect analyses correlating the defect-property modulation in automatically and statistically manners. Figure 1a presents the schematic illustration of the 2H-MoTe_2_ ML sample prepared by (top) mechanical exfoliation and (bottom) subsequently transferred onto a TEM grid, with Mo (Te) denoted by yellow (green) spheres. First, we performed atomic-structural imaging on the as-prepared 2H-MoTe_2_ (hereafter, referred to as pristine 2H-MoTe_2_) at an acceleration voltage of 80 kV. Figure 1b illustrates a high-angle annular dark-field (HAADF)-STEM image of the exfoliated pristine 2H-MoTe_2_ ML, with the bright (dark) contrast regions denoting Te_2_- (Mo) atomic columns. As revealed by atomic structural imaging, the pristine 2H-MoTe_2_ exhibited high crystallinity with no structural degradation, *e.g.*, oxidization of 2H-MoTe_2_.

Based on the Z-contrast imaging principle by STEM,^23^ we identify the brighter (darker) atoms as Te (Mo) with Z = 52 (42). Figure 1b illustrates the wide-view of HAADF-STEM images of pristine 2H-MoTe_2_. The enlarged micrographs from the dotted rectangles denoted by (i) and (ii) in Figure 1b illustrate the defect-free, Perfect (Figure 1b(i)), and incorporated with Te single vacancy, V_Te1_ (red arrow, Figure 1b(ii)), respectively. All V_Te1_ defects are indicated by the red arrows in Figure 1b, and they consistently have the weaker contrast in comparison with pristine Te_2_-atomic column sites. Figure 1b(iii) profiles the intensity along the perfect (defective) lattice indicated by the green (red) rectangle in Figure 1b(i) (Figure 1b(ii)). Herein, we have identified the potential for further defect-engineering to associate defect structure–electrical property modulation with various defect generation sources.

Clearly, there is a significant decrease in the intensity at the V_Te1_ site, which is attributed to one missing Te atom from the Te_2_-atomic column. Based on previous studies, we propose that the identified V_Te1_ is inevitably present during the synthesis or was generated during the mechanical exfoliation.^24,25^ The small concentration of V_Te1_ in the pristine 2H-MoTe_2_ ML is known to induce the weak n-type characteristics, as a result of Fermi-level pinning near the conduction band minimum.^26^ This pinning effect results in lowering of the Schottky barrier height, and it causes an enhancement in the injection of electrons.^27−29^ Therefore, precise control of the V_Te1_ defect is crucial for tailoring the electronic properties exhibition of 2H-MoTe_2_.^20^ After confirming the (i) presence defects (herein, V_Te1_ in small concentration) and (ii) electronic behavior of pristine 2H-MoTe_2_ MLs, the 2H-MoTe_2_ samples on the gold TEM grids subsequently were exposed to disparate external stimuli, *i.e.*, 200 °C-vacuum-annealing and 532 nm-laser-illumination (Figure 1c).

**Figure 2:**
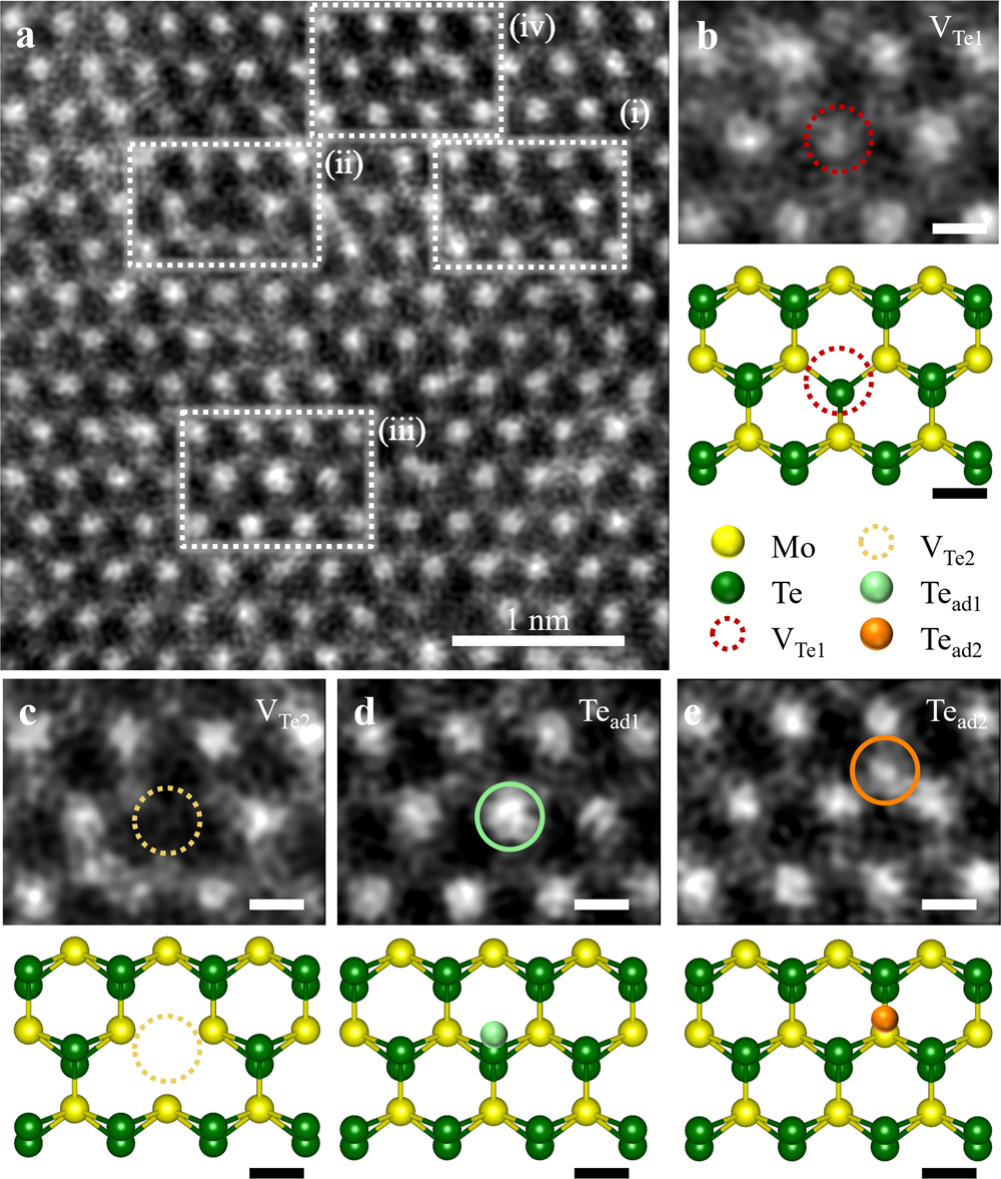
Point defect type exploration for 200 °C-vacuum-annealed 2H-MoTe_2_ (VA 2H-MoTe_2_) ML. (a) Wide-view HAADF-STEM image of VA 2H-MoTe_2_ ML. Typical defects denoted by dotted rectangles (i)–(iv) in (a) represent V_Te1_, V_Te2_, Te_ad1_, and Te_ad2_, respectively. (b)–(e) (Top panels) Enlarged HAADF-STEM micrographs, revealing (i) V_Te1_ (dotted red circle), (ii) V_Te2_ (dotted yellow circle), (iii) Te_ad1_ (solid light-green circle), and (iv) Te_ad2_ (orange solid circle), respectively. (Bottom panels) Corresponding atomic models as top panels; V_Te1_ (V_Te2_) with red (yellow) dotted circle and Te_ad1_ (Te_ad2_) with solid light-green (orange) spheres, respectively. Scale bars: 0.2 nm.

Figure 2a shows a representative HAADF-STEM micrograph of a 200 °C-vacuum-annealed 2H-MoTe_2_ (VA 2H-MoTe_2_) ML. The defective regions accomodating point defects are marked by white dotted rectangles denoting (i) V_Te1_, (ii) Te double vacancies (V_Te2_), (iii) Te adatom on the Te_2_-column (Te_ad1_), and (iv) Te adatom on the Mo-column (Te_ad2_), respectively. Notably, the vacuum-annealing temperature, 200 °C, was judiciously selected to avoid both structural degradation and generation of line defects,^30^ which is beyond the scope of the present study. Based on our STEM inspection, an increase in the species of point defects was identified in VA 2H-MoTe_2_; V_Te1_, V_Te2_, Te_ad1_, and Te_ad2_, compared to pristine 2H-MoTe_2_; V_Te1_.

Figure 2b–e depicts the enlarged HAADF-STEM micrographs from Figure 2a, marked by white dotted rectangles, illustrating specific defect sites of (i) V_Te1_ (red dotted circle), (ii) V_Te2_ (yellow dotted circle), (iii) Te_ad1_ (solid light-green circle), and (iv) Te_ad2_ (solid orange circle), respectively. To confirm the adatom-type in VA 2H-MoTe_2_, we compared the intensity profiles of experimental and simulated defects of Te_ad1_ (Te_ad2_) as presented in Figure S1 (Figure S2). Notably, p-type related defects of the Mo adatom on the Te_2_-column (Mo_ad1_) and Te adatom on the Mo-column (Mo_ad2_) were undetected.^13^ The corresponding atomic models are presented at the bottom for clarity. Because previous studies have suggested that defects *e.g.*, Te vacancies and Te adatoms result in 2H-MoTe_2_ with n-type properties,^30,31^ we contemplate that the VA 2H-MoTe_2_ may exhibit stronger n-type character compared to pristine 2H-MoTe_2_.

**Figure 3:**
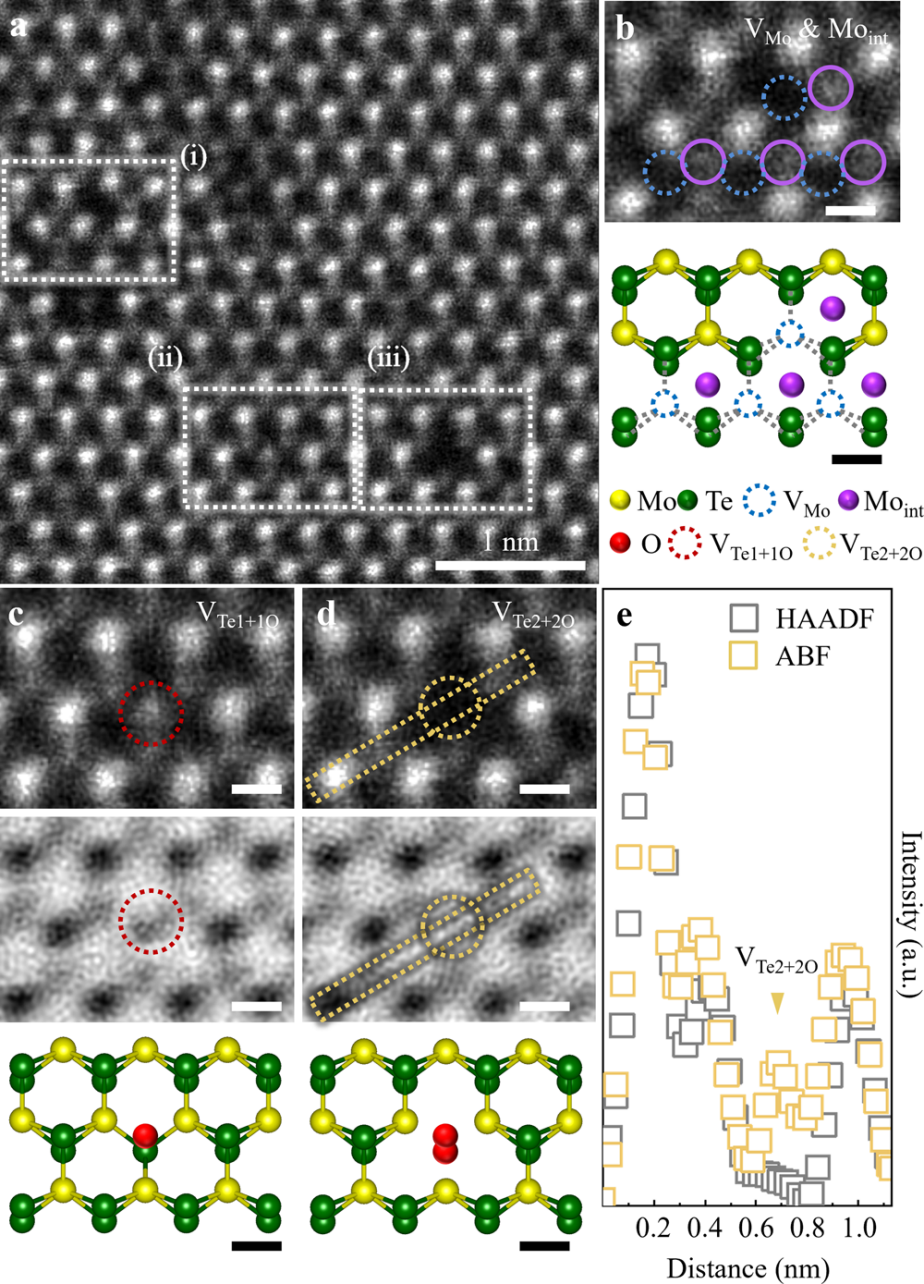
Point defect type exploration for 532 nm-laser-illuminated 2H-MoTe_2_ (LI 2H-MoTe_2_) ML. (a) Wide-view HAADF-STEM image of LI 2H-MoTe_2_ ML. Typical defects denoted by white dotted rectangles (i)–(iii) in (a) represent (i) V_Mo_ coupled with Mo_int_, (ii) V_Te1+1O_, and (iii) V_Te2+2O_, respectively. (b) (Top panel) Enlarged HAADF-STEM micrographs in (a), revealing (i) V_Mo_ (dotted blue circle) and Mo_int_ (solid purple circle). (Bottom panel) Corresponding atomic model as top panel; V_Mo_ (Mo_int_) with dotted blue circle (solid purple sphere). (c, d) (Top panels) Enlarged HAADF-STEM micrographs in (a) revealing (ii) V_Te1+1O_ (dotted red circle) and (iii) V_Te2+2O_ (dotted yellow circle), respectively. (Middle panels) Corresponding ABF-STEM as top panels illustrating enhanced atomic contrast attributed to oxygen adsorption at each V_Te1_ and V_Te2_ sites. (Bottom panels) Corresponding atomic configurations with oxygen atom (red sphere). Scale bars, 0.2 nm. (e) Intensity profiles for HAADF- (open gray squares) and ABF- (open yellow squares) regions extracted along the dotted diagonal yellow rectangles in the top and bottom panels in (d), respectively. Note that oxygen contrast is detectable only in ABF-STEM (yellow arrow).

Next, we investigated the atomic structure of 532 nm-laser-illuminated 2H-MoTe_2_ (LI 2H-MoTe_2_) MLs. Figure 3a is a representative HAADF-STEM image of LI 2H-MoTe_2_. The defective regions with point defects are marked by white dotted rectangles denoting (i) Mo single vacancy (V_Mo_) coupled with Mo interstitial (Mo_int_), (ii) one oxygen atom adsorbed/chemisorbed at the V_Te1_ site (V_Te1+1O_), and (iii) two oxygen atoms adsorbed/chemisorbed at the V_Te2_ site (V_Te2+2O_), respectively. Figure 3b shows an enlarged HAADF-STEM micrograph from the dotted rectangle (i) in Figure 3a. By comparing the intensity profiles between the experimental and simulated HAADF-STEM image analyses (Figure S3), the representative defects in Figure 3b were confirmed as V_Mo_ (blue dotted circle) and Mo_int_ (solid purple circle) pair. The corresponding atomic model is also shown at the bottom, where V_Mo_ (Mo_int_) defects are denoted by the blue (purple) dotted circles (spheres). The concurrent observation of V_Mo_ and Mo_int_ pair implies that a Mo atom in a regular unit cell is shifted about a half-unit-cell-length by a 532 nm-laser. This type of defect pair has not been observed so far, despite their (V_Mo_ and Mo_int_) p-type impact on the electronic property of LI 2H-MoTe_2_. Furthermore, Te interstitial (Te_int_), which contributes to the n-type character, is undetected.^30^

As previously mentioned, early research works had indicated that a few-layer-thin 2H-MoTe_2_ exposed to a laser (particularly, of 532 nm wavelength) exhibits p-type doping due to adsorption/chemisorption of oxygen atoms at the V_Te_ sites.^31^ However, because of issues related to sample thickness as well as laser-induced sample damage, direct visualization and study of oxygen-related defects in LI 2H-MoTe_2_ MLs has been challenging.^32^ In addition, the oxygen atom exhibits a low atomic number (Z = 8) which is nearly impossible to detect by the highly Z-sensitive conventional HAADF-STEM detector.^23^ Moreover, further complications in identifying oxygen-related defects arise when the oxygen atoms coexist with heavy atomic elements *e.g.*, Te (Z = 52) and Mo (Z = 42). For simplicity and the direct interpretation of oxygen-related defects, the 2H-MoTe_2_ samples prepared for this study were entirely composed of “MLs”. Here, the 2H-MoTe_2_ ML would be much more photon-sensitive than multilayered 2H-MoTe_2_, and to achieve minimal structural degradation induced by laser-induced heating (light absorption), the exposure time was set to be 5 s, combined with defocus illumination (see more details in Figure S4). With the combination of an optimal laser-illumination setup and ML-targeted analysis, we could achieve the distinctive point defect identification and further deep learning-based point defect examinations, which will be discussed later.

For V_Te_-related defects, we simultaneously employed (i) an HAADF detector to collect scattered electron signals by heavy atoms, Mo (Te), and (ii) a light-element sensitive annular bright field (ABF) detector to collect scattered electron signals from oxygen atoms.^33,34^Figure 3c,d shows enlarged (top) HAADF- and (middle) ABF-STEM images from the white dotted rectangles ((ii) and (iii)) in Figure 3a. The corresponding atomic models are presented at the bottom for clarity. From a close-up view, a slight increase in atomic contrast can be seen at the V_Te_ sites (red and yellow dotted circles) in the ABF-STEM image. This contrast is more pronounced in the ABF-STEM of Figure 3d, where two Te atoms are missing (yellow dotted circle). The appearance of such a weak contrast in the ABF- but not HAADF-STEM image signatures is due to the presence of a light element (possibly, oxygen atom) adsorbed/chemisorbed at the V_Te_ site.

To verify our hypothesis, we performed a series of simulated HAADF- and ABF-STEM analyses to discern the presence of oxygen atoms adsorbed/chemisorbed at the V_Te_ sites, as illustrated in Figure S5. Here, we considered all possible defects related to the V_Te_ site: (i) V_Te1_, (ii) V_Te1+1O_, (iii) V_Te2_, (iv) one oxygen atom adsorbed/chemisorbed at the V_Te2_ site (V_Te2+1O_), and (v) V_Te2+2O_. The simulation parameters were set comparable to those used in our experiment. (See more details in Methods). Subsequently, we compared the experimental and simulated HAADF- and ABF-STEM images of the V_Te_ sites and their corresponding intensity profiles (Figure S6). We confirm the defective Te_2_-colum sites in Figure 3c,d to comprise V_Te1+1O_ (dotted red circle) and V_Te2+2O_ (dotted yellow circle).

Figure 3e profiles the corresponding HAADF- and ABF-STEM intensities extracted from Figure 3d along the regions marked by yellow dotted rectangles. As expected, the ABF-STEM intensity profile (open yellow square) illustrates the presence of a weak contrast at the V_Te2_ site which is absent in the HAADF-STEM intensity profile (open gray square). This clearly confirms the presence of the V_Te2+2O_ and V_Te2+1O_ defects observed in our experiment, which coincides with the previous report.^31^ To ascertain our conclusion, we further analyzed and compared ABF-STEM images obtained from VA 2H-MoTe_2_ and LI 2H-MoTe_2_ MLs (Figure S7). We could not observe oxygen-related weak contrast at the V_Te2_ site in the ABF-STEM of the VA 2H-MoTe_2_ ML, while clear contrast was observed at the V_Te2_ site in the ABF-STEM of the LI 2H-MoTe_2_. The direct visualization of oxygen atoms adsorbed/chemisorbed at the V_Te_ in this study further corroborates the presence of the oxygen-induced p-type doping mechanism in LI 2H-MoTe_2_. (For an alternative strategy for p-doping of 2H-MoTe_2_, see Figure S8 for oxygen plasma-treated (PT) 2H-MoTe_2_ MLs.)

**Figure 4:**
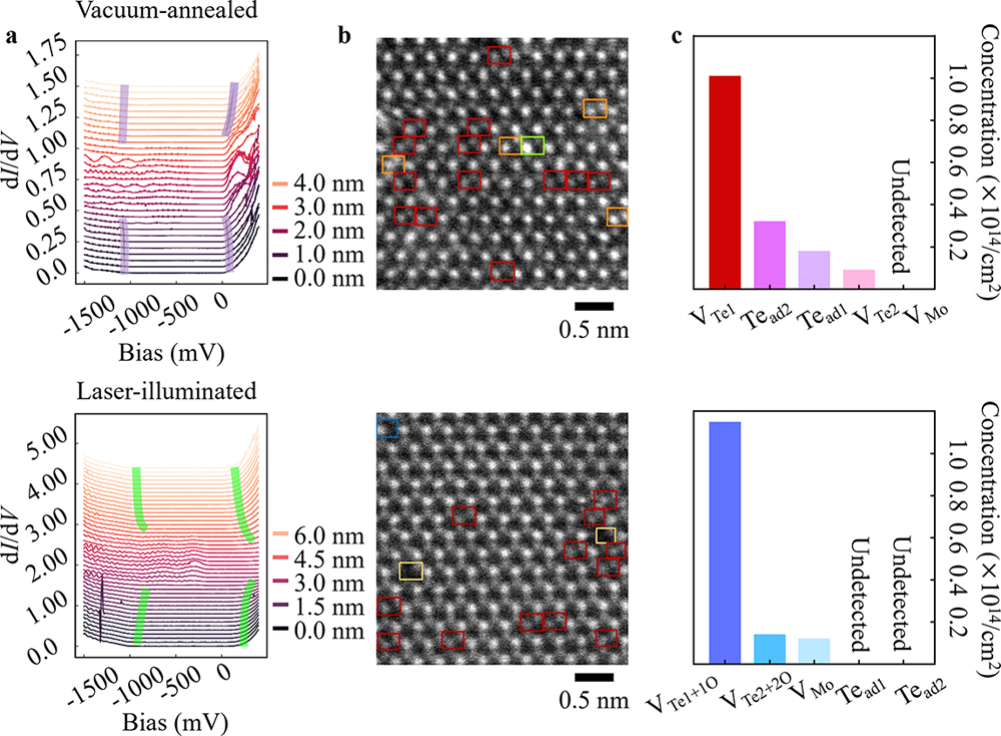
Quantification of point defect species and point defect classification in VA 2H-MoTe_2_ and LI 2H-MoTe_2_. (a) (Top)–(Bottom) Variations in the d*I*/d*V* line spectra by STS near the point defects such as (i) Te_ad1_ or V_Te1_ in VA 2H-MoTe_2_ and (ii) V_Mo_, V_Te1+1O_ in LI-2H-MoTe_2_, respectively. A shift in the LDOS toward the occupied (unoccupied) states as shown by purple (green) lines depict n-type (p-type) character. The legends denote the distance of the tip for the STS measurement from the defect site. The 200 °C vacuum annealing transpires a Te vacancy (Te adatom) inducing a strong n-type character. Conversely, the 532 nm-laser-illumination transpires oxygen adsorbed/chemisorbed at the Te vacancy site (V_Te_) inducing strong p-type character. (b) (Top, bottom) Estimated point defect classification results by deep learning for VA 2H-MoTe_2_ and LI 2H-MoTe_2_ MLs, respectively. The Perfect types are not indicated, though they were classified by deep learning. Color codes are all the same as in Figures 2 and 3 for defect species. Note that V_Mo_ was undetected in VA 2H-MoTe_2_ and Te_ad1_ (Te_ad2_) was undetected in LI 2H-MoTe_2_. (c) (Top, Bottom) Statistical point defect classification by deep learning in VA 2H-MoTe_2_ and LI 2H-MoTe_2_ MLs respectively to correlate point defect-electrical property modulations. V_Te1_ (red), V_Te2_ (light-magenta), Te_ad1_ (light-purple), and Te_ad2_ (light-pink) for VA 2H-MoTe_2_; V_Te1+1O_ (blue), V_Te2+2O_ (light-blue), and V_Mo_ (sky-blue) for LI 2H-MoTe_2_.

To clarify the defect-modulated properties, we investigated the electrical transport of the VA 2H-MoTe_2_ and LI 2H-MoTe_2_ MLs by scanning tunneling spectroscopy (STS) as depicted in Figure 4. For n-type doping of 2H-MoTe_2_, vacuum-annealing was reported to be effective, revealed by device fabrication,^35^ or by STS measurements on the defective surfaces.^30^ In contrast to past studies that performed STS measurements near defects from a relatively large sample area and which may be prone to errors, we inspected STS near the defective site with a 1 nm step to tightly monitor the defect-property correlation. Figure 4a (top) displays a variation in d*I*/d*V* extracted near defective sites (0.0 to 4.0 nm with 1.0 nm increment) in VA 2H-MoTe_2_. A closer observation of the LDOS reveals a substantial shift toward the occupied states (purple guidelines) indicating the presence of donor/n-type dopants (strong n-type 2H-MoTe_2_). Previous works indicated that air-doping^26^ and oxygen intercalation^27,36^ are effective methods for p-type doping of 2H-MoTe_2_. However, since 2H-MoTe_2_ is structurally vulnerable compared to other TDMCs,^13^ both strategies may not be free of structural degradation of 2H-MoTe_2_ by H_2_O or oxidization. Furthermore, the electrical property results obtained were a reflection of the overall performance of FETs and not a precise study of the electronic property of each defective site. However, we inspected STS near the defective site with a 1.5 nm step to tightly monitor the defect-property modulations.

Figure 4a (bottom) shows variation in d*I*/*dV* extracted near defective sites (0.0 to 6.0 nm with 1.5 nm increment) in LI 2H-MoTe_2_. We noticed a strong shift of the LDOS toward the unoccupied states (green guide lines) signifying the presence of acceptor/p-type dopants (strong p-type 2H-MoTe_2_). Through Figure 4a,b, we confirmed the (i) strong n-type character of VA 2H-MoTe_2_ and (ii) strong p-type character LI 2H-MoTe_2_ revealed by STS measurements. We can strongly mention that these properties are directly correlated with point defects: (i) V_Te1_, V_Te2_, Te_ad1_, and Te_ad2_ for n-type (confirmed by Figure 2 and Figures S1–S2) and (ii) V_Mo_, Mo_int_, V_Te1+1O_, and V_Te2+2O_ for p-type (confirmed by Figure 3, Figure S3, and Figures S5–S7). Although HAADF-STEM based previous studies reported representative atomic defects of Te-vacancy and in n-type doped 2H-MoTe_2_,^30^ direct probing of the local electronic properties of such defects using STS is still limited. Further reports only mentioned that these defects are responsible for the dominant n-type characteristics.^27,28,35^ For p-type 2H-MoTe_2_, oxygen atoms adsorbed/chemisorbed at vacant sites were claimed for possible candidates.^26,27,36^ These studies did not present detailed defect structures, presumably suggesting possible point defect types combined with device characterization or theoretical approaches. Obviously, we have identified point defect species in VA 2H-MoTe_2_ and LI 2H-MoTe_2_ MLs by comparing experimental and simulated intensity profiles in Figures 2 and 3. However, these approaches limit the statistical analyses of the various point defect species and further the association of defect-property units.

As an alternative, we incorporated a deep learning algorithm to identify and quantify the defect species and their overall distribution in the VA 2H-MoTe_2_ and/or LI 2H-MoTe_2_ MLs. Here, the deep learning models were adopted to (i) recognize and crop hexagonal cells^37^ and then (ii) classify point defect species in each unit cell cropped from the hexagonal cells. Briefly, (i) faster R-CNN (region-based CNN) was utilized to eventually detect and predict the unit cell locations optimal for object detection, as reported in our earlier study.^37^ Next, we adopted (ii) the Fully Convolutional Network (FCN), which is widely used to classify the point defect species. Figure S9 illustrates the point defect analysis workflow by FCN. From the Faster R-CNN output, the FCN model predicts the species of point defects in a unit cell: Te on-site and Mo on-site defects, respectively. With the combination of two atomic-column point defect classifications, eventual point defect species are determined. Also, Figure S10 shows the basic process based on deep learning: model description, training results for each atomic-column point defect classification of FCN models, and the point defect classification results of simulated images. See more details in Supplementary Text 5 and Figures S9–S10.

Figure 4b demonstrates the application of our deep learning algorithm to the experimental HAADF-STEM images of (top) VA 2H-MoTe_2_ and (bottom) LI 2H-MoTe_2_ MLs, respectively. Four characteristic defects were identified: V_Te1_ (red), V_Te2_ (yellow), Te_ad1_ (light-green), and Te_ad2_ (orange) in VA 2H-MoTe_2_, and three characteristic defects were identified: V_Te1+1O_ (red), V_Te2+2O_ (yellow), and V_Mo_ (blue) in LI 2H-MoTe_2_. As we expressed in Figure 3d,e, we could identify the V_Te2+2O_ by comparing HAADF- and ABF-STEM image intensities (Figure 3e). Also, according to previous study,^31^ regardless of the ambient air or O_2_ gas atmosphere, 2H-MoTe_2_ was modulated to a p-type semiconductor induced by oxygen atoms adsorbed/chemisorbed at the V_Te_ sites. In this study, the dominant reactant species for p-doping were “oxygen” atoms, confirmed by cross-sectional energy-dispersive X-ray spectroscopy mapping and in-plane STEM image analysis. Since we detected the oxygen atoms at Te vacancies (Figure 3d,e) and set the identical laser-illumination condition as reported,^31^ we could determine oxygen atoms adsorbed/chemisorbed at the V_Te_ sites within the crystal matrix, even in HAADF-STEM images.

To clarify the point defects in each defect-engineered 2H-MoTe_2_, we analyzed pristine 2H-MoTe_2_, where V_Te1_ defects in low concentration were also confirmed (Figure S11). Through the benchmark, the analytical accuracy of our deep learning-based defect analyzer reaches up to 99.3%, and the detailed methodology is described in Figures S12–S15. (Also, see more details in Figure S14 for PT 2H-MoTe_2_.) Strikingly, p-type (n-type) related V_Mo_ defects (Te_ad1_ and Te_ad2_ defects) were undetected in the pristine 2H-MoTe_2_ in Figure S12 (Figure S13). These indicate that the defect identification with the deep learning algorithm accurately captures the defect responsible for the electronic properties of defect-engineered 2H-MoTe_2_. Previously, the impacts of point defects in 2D materials were examined either theoretically^10,13,16^ or experimentally,^11−14,27−37^ though the defect-generation strategies and corresponding defect-type formation were hereto unspecified quantitatively. Our results obtained by a deep learning algorithm pinpointed the impact of defect-engineering methods on the electronic properties of 2H-MoTe_2_; “certain” external stimuli can transpire “specific defect-types” manifesting electric properties. By applying our deep learning models, the categorization of point defect-oriented defect-engineering can be realized.

There is still a need to address the “degree of contribution” of each defect species to the properties in crystal systems, as it remains unexplored. First, to clearly define the defects in VA 2H-MoTe_2_ and LI 2H-MoTe_2_, we examined the defects in pristine 2H-MoTe_2_ (Figure S11); V_Te1_ with concentration of 0.58 × 10^14^/cm^2^ (total analyzed area of 1.38 × 10^15^/cm^2^, 13.8 nm^2^). The defect concentration of pristine 2H-MoTe_2_ was reported as “0.48/100 nm^2^ (0.48 × 10^12^ cm^2^)”;^20^ however, this report did not discriminate the Te vacancy and Te adatom types, which could not further tailor the concentration of defect-type and corresponding property units. Here, we could quantify the defect species in a statistical approach as presented in Figure 4c (top) VA 2H-MoTe_2_ and (bottom) LI 2H-MoTe_2_ MLs (excluding perfect ones), respectively. The *x*-axis (*y*-axis) indicates the point defect species (defect concentrations in units of “× 10^14^/cm^2^”).

It is evident that 200 °C-vacuum-annealing results in V_Te1_ (red), V_Te2_ (light-magenta), Te_ad1_ (light-purple), and Te_ad2_ (light-pink). The most dominant defect species was “V_Te1_” with the concentration being 1.01 × 10^14^/cm^2^. The concentration distribution of Te_ad2_, Te_ad1_, and V_Te2_ defects were in the order of 0.32 × 10^14^/cm^2^, 0.18 × 10^14^/cm^2^, and 0.09 × 10^14^/cm^2^, respectively (total analyzed area of 2.18 × 10^15^ cm^2^ (21.8 nm^2^)). We focused on analyzing “ML” 2H-MoTe_2_ and successfully achieved quantitative categorization of the point defect type and their concentrations. For multilayered 2H-MoTe_2_, one can notice the (i) vacancy or (ii) adatom site; however, due to the stacking order, defect type determination is challenging, *e.g.*, (i) Mo vacancies *vs* Te vacancies or (ii) Te adatom on Mo-column *vs* Te_2_-column. Furthermore, judicious selection of annealing temperature achieved minimal structural degradation of 2H-MoTe_2_ MLs, which enabled the deep learning application to the atomic-structural images.

For the LI 2H-MoTe_2_, the most dominant defect species was “V_Te1+1O_” with the concentration being 1.15 × 10^14^/cm^2^. The concentration distribution of V_Te2+2O_ and V_Mo_ defects were in the order of 0.14 × 10^14^/cm^2^ and 0.12 × 10^14^/cm^2^, respectively (total analyzed area of 4.25 × 10^15^ cm^2^ (42.5 nm^2^)). Though we analyzed HAADF-STEM images to identify V_Te1+1O_ and V_Te2+2O_ by FCN, both V_Te1+1O_ and V_Te2+2O_ were affirmed as illustrated in ABF-STEM images in Figure 3 and Figures S4–S6. Here, the V_Mo_ was directly detected since we confined to analyze 2H-MoTe_2_ ML; only one Mo atom exists in the unit cell of 2H-MoTe_2_ ML. From the previous reports, (i) oxygen atoms adsorbed/chemisorbed at the V_Te_ or V_Mo_ and (ii) V_Mo_ were revealed to dope 2H-MoTe_2_ to p-type by visible-laser-illumination.^31^ Although the study straightforwardly correlated defect-property exhibition suggesting representative point defects, statistical approach was still constrained since “bilayer” 532 nm-laser-illuminated 2H-MoTe_2_ was analyzed. However, we could unambiguously categorize and reveal the defect-types and their concentrations by inspecting “ML” LI 2H-MoTe_2_, which has not been realized so far.

## Conclusion

We synergistically integrated the complementary strengths of (i) low-voltage 5^th^-order *C*_*s*_-corrected HAADF- and ABF-STEM imaging with (ii) atomic-scale simulations and (iii) a deep learning algorithm to explore various defect species in pristine 2H-MoTe_2_, VA 2H-MoTe_2_, and LI 2H-MoTe_2_ MLs. In particular, by combining deep learning classification and quantification of point defects alongside STS analysis, we demonstrate that 200 °C-vacuum-annealing creates Te-vacancies and Te-adatoms with strong n-type characteristics while 532 nm-laser-illumination promotes oxygen atoms adsorption/chemisorption at the V_Te_ sites exhibiting strong p-type characteristics. Our deep learning algorithm performs efficiently and accurately in classifying on-site point defects in defect-engineered-2H-MoTe_2_ MLs. Still, further improvements should be conducted (as shown in Figure S15) and explored for randomly distributed defects *e.g.*, interstitial defects. All in all, our research not only suggests a strong foundation for rudimental understanding of defect-engineered 2H-MoTe_2_ and other 2D materials but also provides a generic platform where deep learning can be combined with atomic simulations and electron microscopy to probe multiscale processes associated with complex materials phenomena.

## Methods

### TEM Sample Preparation of 2H-MoTe_2_ MLs

First, blue tape was used to exfoliate ML 2H-MoTe_2_ from a bulk sample purchased from *2D Semiconductor*. We used 2-propanol (isopropanol) to clean the SiO_2_ (∼290 nm)/Si substrate and then rinsed it with deionized (DI) water. The blue tape containing ML-thick 2H-MoTe_2_ was carefully brought into contact with the SiO_2_/Si substrate and 2H-MoTe_2_ flakes were transferred onto the SiO_2_ surface owing to the capillary force between the 2H-MoTe_2_ flakes and SiO_2_. The poly(methyl 2-methylpropenoate) [*i.e.*, poly(methyl methacrylate), PMMA] coating was then spun onto the SiO_2_/Si substrate with 2H-MoTe_2_ flakes at 4300 rpm for approximately 60 s and then cured at 100 °C for 2 min. The ML 2H-MoTe_2_ sample was detached from the substrate by etching in 10 wt % hydrofluoric acid to dissolve the SiO_2_ layer, leaving the PMMA-coated 2H-MoTe_2_ film floating. The PMMA-coated sample was carefully rinsed twice with DI water. Next, a Quantifoil holey carbon 200-mesh TEM grid was used to fish it out of the DI water. The TEM grid was then soaked with the PMMA-coated sample in acetone for approximately 2–3 h to remove the PMMA coating and obtain clean flakes. To remove any residual polymer, we vacuum annealed the sample under mild conditions at 150 °C to obtain clean 2H-MoTe_2_ MLs.

### Point Defect Control Process of 2H-MoTe_2_ MLs

After preparing three mechanically exfoliated monolayer 2H-MoTe_2_ samples, vacuum-annealing was performed at 200 °C for 2 h on the first sample under pressure of 7.5 × 10^–7^ Torr. The second sample was exposed to 532 nm-laser-illumination with a power of 20 mW and spot size of ∼5 μm.^31^ With the identical experimental condition, *i.e.*, laser source and power,^31^ we illuminated the monolayer 2H-MoTe_2_ exposure time of 5 s. Also, if we focused the laser beam on the specimen (even for the bulk sample), the sample was damaged as illustrated in Figure S4. Note that the distance between the laser-source and the TEM-sample was 40 cm, with no Neutral Density (ND) filter. Therefore, to mildly illuminate a broad range of the pristine 2H-MoTe_2_, we (i) inserted an ND filter between the laser-source and the TEM sample to reduce the light quantity with a distance of 30 cm (*d*_*1*_) and (ii) adjusted the distances between the ND filter and the TEM sample of 30 cm (*d*_*2*_). The *d*_*1*_ + *d*_*2*_ (60 cm) determines the defocused-laser condition to the TEM sample (distance-in-focus of 40 cm in Figure S4a), with mild defect generations by an ND filter (Figure S4b). These three factors, *i.e.*, reduced exposure time, reduced light quantity of the laser source, and a defocused laser, are essential because monolayer 2H-MoTe_2_ is highly photon-sensitive compared to bulk 2H-MoTe_2_. The third sample was exposed to oxygen plasma for 10 s at room temperature in an inductively coupled plasma system equipped with a 13.56 MHz microwave (miniplasma-station, Plasmart) at a pressure of 20 Pa and oxygen flow rate of 30 sccm.

### Atomic-Scale Imaging and Simulation

We conducted atomic-scale image analysis using 5^th^-order *C*_*s*_-corrected STEM (JEM-ARM200F, JEOL, Japan) at the Materials Imaging & Analysis Center of POSTECH and incorporated a 5^th^-order *C*_*s*_-corrector (ASCOR, CEOS GmbH, Germany) operated at an acceleration voltage of 80 kV. We intentionally adopted low-voltage imaging to minimize the electron beam-induced damage to the sample during STEM imaging. We optimized the experimental conditions for atomic-scale imaging: (i) camera length of 8 cm to acquire HAADF (inner–outer angle of 54.0–216.0 mrad) and ABF (inner–outer angle of 13.5–27.0 mrad) images, (ii) probe size of 9 C (current density of ∼4.5 pA/cm^2^), and (iii) with a 40 μm condenser aperture, corresponding to a convergence angle of 27 mrad. We performed simulations using Material Studio,^38^ Vesta,^39^ and Dr. Probe software,^40^ which allowed us to construct crystal models based on the available crystallographic information files. We generated the training data set with experimental conditions identical to the HAADF- (ABF-)STEM imaging conditions above. For training data set generation, we added Poisson (Gaussian) noise values of 5 ≤ λ ≤ 20 (μ = 20) to each simulation image. Obviously, for compatibility of experimental and simulation images, we normalized the experimental data format with simulation images.

### Classification of Atomic Defects by Deep Learning

We separated the analytic phases in two steps (Figure S9): deep learning (DL) processing and postprocessing. The DL processing utilizes three deep learning networks for (i) unit cell detection, (ii) Te on-site defect identification, and (iii) Mo on-site defect identification. For the unit cell detection, the developed deep learning model was used, based on the convolutional neural network (CNN) as reported in our previous studies.^37^ Here, the model Faster R-CNN is trained to detect hexagonal cells, and then the unit cell is detected by further unit cell cropping. For point defect identification, the FCN model is widely used for point defect segmentation. From the defect-classified results of the Te on-site or Mo on-site defect in a unit cell, each image is fed to the Faster R-CNN. With the combination of Te on-site or Mo on-site defect-classified results, the final defect species, *e.g.*, V_Te1_ from Te on-site and V_Mo_ from Mo on-site, result in V_Te1_+V_Mo_. For each point defect classification model, we trained two networks, each segmenting point defects in the Te_2_-column and Mo-column, using 2000 simulated images for 300 epochs (see Figure S10 for the structure and description of the FCN model). Unit cell areas detected from faster R-CNN are applied to each FCN model. Te on-site (Te defect) defect types and Mo on-site (Mo defect) defect types in the same unit cell area are calculated as final defects through combination. Point defect types—vacancy and adatoms—are classified with high accuracies as reported in our results. Here, we augmented the training data set by varying noise, brightness, and crop size.

### Device Fabrication

ML 2H-MoTe_2_ flakes were mechanically exfoliated onto SiO_2_ (300 nm; back-gate oxide thickness)/p^+^-Si substrates using the standard Scotch tape technique. The bulk 2H-MoTe_2_ crystals, purchased from *2D Semiconductors* with a specified purity of 99.9999% and a defect concentration of ∼10^12^ cm^–2^, served as the source material. Followed by exfoliation, the freshly obtained 2H-MoTe_2_ MLs underwent a 3 h immersion in acetone to eliminate any residual tape residues.

For the FET fabrication process, a layer of PMMA (electron beam (e-beam) resist, MicroChem) was spin-coated at 4000 rpm for 1 min, followed by a 30 s bake at 180 °C. Subsequently, e-beam lithography was employed to define the channel dimension (length of ∼1 μm and width of ∼3 μm) and the source/drain contacts. Metallization was achieved through e-beam evaporation, involving the deposition of a 5 nm Ti layer at a rate of 0.5 Å/s, followed by a 50 nm Au layer at a rate of 1.0 Å/s, performed under a vacuum of approximately ∼10^–7^ Torr. A 3 h acetone lift-off process was then conducted.

### Electrical Characterization of Vacuum-Annealed and Laser-Illuminated 2H-MoTe_2_

We used the commercial low-temperature scanning tunneling microscope (Unisoku, Ltd., Japan) for the STS measurements. n-type or p-type was prepared by mechanical exfoliation and treated with similar conditions to those for STEM study, exposed to 200 °C-vacuum-annealing and 532 nm-laser-illumination. The samples were then transferred to ultrahigh vacuum (UHV, base pressure < 10^–10^ Torr), and the single crystals were cleaved to expose the clean surface. We then used the electrochemically etched W (tungsten) tip after the electron bombardment heating. We performed STS measurements at low temperature (78 K) and precisely positioned the probing tip to the laser-illuminated area using working-distance optical microscopy.

## Data Availability

The codes and data set used in this study are available from the corresponding author upon reasonable request. Also, our GUI is publicly available on GitHub https://github.com/wormschu/FCN-Detection-based-MoTe-defect-analysis.

## Supporting Information Available

Figures S1–S15 and Supporting Texts 1–5 detailing (i) experimental and simulation HAADF-STEM analyses of defects in 200 °C-vacuum-annealed 2H-MoTe_2_ ML; (ii) experimental and simulation HAADF-(ABF-)STEM analysis of defects in 532 nm-laser-illuminated 2H-MoTe_2_ ML; (iii) a comparison between ABF-STEM image analyses of 200 °C-vacuum-annealed and 532 nm-laser-illuminated 2H-MoTe_2_ MLs; (iv) experimental HAADF-(ABF-)STEM analysis of oxygen plasma-treated 2H-MoTe_2_ ML; and (v) deep learning-based inspection of point defects in pristine and defect-engineered 2H-MoTe_2_ MLs (PDF)

## References

[ref1] Hong J.; Jin C.; Yuan J.; Zhang Z. Atomic Defects in Two-Dimensional Materials: From Single-atom Spectroscopy to Functionalities in Opto-/Electronics, Nanomagnetism, and Catalysis. Adv. Mater. 2017, 29, 1606434 10.1002/adma.201606434. 28295728

[ref2] Goodnick S. M.; Gann R. G.; Ferry D. K.; Wilmsen C. W.; Krivanek O. L. Surface roughness induced scattering and band tailing. Surf. Sci. 1982, 113, 233–238. 10.1016/0039-6028(82)90591-X.

[ref3] Goodnick S. M.; Gann R. G.; Sites J. R.; Ferry D. K.; Wilmsen C. W.; Fathy D.; Krivanek O. L. Surface roughness scattering at the Si-SiO_2_ interface. J. Vac. Sci. Technol. 1983, 1, 803–808. 10.1116/1.582696.

[ref4] Geim A. K.; Novoselov K. S. The rise of graphene. In Nanoscience and technology: a collection of reviews from Nature Journals; World Scientific: 2010; pp 11–19.

[ref5] Zhang M. Y.; Wang Z. X.; Li Y. N.; Shi L. Y.; Wu D.; Lin T.; Zhang S. J.; Liu Y. Q.; Liu Q. M.; Wang J.; Dong T.; Wang N. L. Light-Induced Subpicosecond Lattice Symmetry Switch in MoTe_2_. Phys. Rev. X 2019, 9, 021036 10.1103/PhysRevX.9.021036.

[ref6] Schwierz F.; Pezoldt J.; Granzner R. Two-dimensional materials and their prospects in transistor electronics. Nanoscale 2015, 7, 8261–8283. 10.1039/C5NR01052G. 25898786

[ref7] Mitta S. B.; Choi M. S.; Nipane A.; Ali F.; Kim C.; Teherani J. T.; Hone J.; Yoo W. J. Electrical characterization of 2D materials-based field-effect transistors. 2D Mater. 2021, 8, 012002 10.1088/2053-1583/abc187.

[ref8] Zheng Y.; Gao J.; Han C.; Chen W. Ohmic Contact Engineering for Two-Dimensional Materials. Cell Rep. Phys. Sci. 2021, 2, 100298 10.1016/j.xcrp.2020.100298.

[ref9] Jiang J.; Xu T.; Lu J.; Sun L.; Ni Z. Defect Engineering in 2D Materials: Precise Manipulation and Improved Functionalities. Research 2019, 10.34133/2019/4641739. PMC6944491 31912036

[ref10] Lin Z.; Carvalho B. R.; Kahn E.; Lv R.; Rao R.; Terrones H.; Pimenta M. A.; Terrones M. Defect engineering of two-dimensional transition metal dichalcogenides. 2D Mater. 2016, 3, 022002 10.1088/2053-1583/3/2/022002.

[ref11] Chen J.; Shan Y.; Wang Q.; Zhu J.; Liu R. P-type laser-doped WSe_2_/MoTe_2_ van der Waals heterostructure photodetector. Nanotechnol. 2020, 31, 295201 10.1088/1361-6528/ab87cd. 32268302

[ref12] Ryder C. R.; Wood J. D.; Wells S. A.; Hersam M. C. Chemically Tailoring Semiconducting Two-Dimensional Transition Metal Dichalcogenides and Black Phosphorus. ACS Nano 2016, 10, 3900–3917. 10.1021/acsnano.6b01091. 27018800

[ref13] Seo S.-Y.; Yang D.-H.; Moon G.; Okello O. F. N.; Park M. Y.; Lee S.-H.; Choi S.-Y.; Jo M.-H. Identification of point defects in atomically thin transition-metal dichalcogenide semiconductors as active dopants. Nano Lett. 2021, 21, 3341–3354. 10.1021/acs.nanolett.0c05135. 33825482

[ref14] Xiong Z.; Zhong L.; Wang H.; Li X. Structural Defects, Mechanical Behaviors, and Properties of Two-Dimensional Materials. Mater. 2021, 14, 1192 10.3390/ma14051192. PMC7961825 33802523

[ref15] Wang X.; Sun Y.; Liu K. Chemical and structural stability of 2D layered materials. 2D Mater. 2019, 6, 042001 10.1088/2053-1583/ab20d6.

[ref16] Bhimanapati G. R.; Lin Z.; Meunier V.; Jung Y.; Cha J.; Das S.; Xiao D.; Son Y.; Strano M. S.; Cooper V. R.; Liang L.; Louie S. G.; Ringe E.; Zhou W.; Kim S. S.; Naik R. R.; Sumpter B. G.; Terrones H.; Xia F.; Wang Y.; et al. Recent Advances in Two-Dimensional Materials beyond Graphene. ACS Nano 2015, 9, 11509–11539. 10.1021/acsnano.5b05556. 26544756

[ref17] Okello O. F. N. O.; Doh K.-Y.; Kang H. S.; Song K.; Kim Y.-T.; Kim K. H.; Lee D.; Choi S.-Y. Visualization of Transition Metal Decoration on h-BN surface. Nano Lett. 2021, 21, 10562–10569. 10.1021/acs.nanolett.1c02198. 34618461

[ref18] Leiter R.; Li Y.; Kaiser U. *In-situ* formation and evolution of atomic defects in monolayer WSe_2_ under electron irradiation. Nanotechnol. 2020, 31, 495704 10.1088/1361-6528/abb335. 32946426

[ref19] Li W.; Field K. G.; Morgan D. Automated defect analysis in electron microscopic images. npj Comput. Mater. 2018, 4, 36 10.1038/s41524-018-0093-8.

[ref20] Elibol K.; Susi T.; Argentero G.; Monazam M. R. A.; Pennycook T. J.; Meyer J. C.; Kotakoski J. Atomic Structure of Intrinsic and Electron-Irradiation-Induced Defects in MoTe_2_. Chem. Mater. 2018, 30, 1230–1238. 10.1021/acs.chemmater.7b03760. 29503509 PMC5830698

[ref21] Frey N. C.; Akinwande D.; Jariwala D.; Shenoy V. B. Machine Learning-Enabled Design of Point Defects in 2D materials for Quantum and Neuromorphic Information processing. ACS Nano 2020, 14, 13406–13417. 10.1021/acsnano.0c05267. 32897682

[ref22] Maksov A.; Dyck O.; Wang K.; Xiao K.; Geohegan D. B.; Sumpter B. G.; Vasudevan R. K.; Jesse S.; Kalinin S. V.; Ziatdinov M. Deep learning analysis of defect and phase evolution during electron beam-induced transformations in WS_2_. npj Comput. Mater. 2019, 5, 12 10.1038/s41524-019-0152-9.

[ref23] Tang Y. L.; Zhu Y. L.; Ma X. L. On the benefit of aberration-corrected HAADF-STEM for strain determination and its application to tailoring ferroelectric domain patterns. Ultramicroscopy 2016, 160, 57–63. 10.1016/j.ultramic.2015.09.014. 26452195

[ref24] Zhou W.; Zou X.; Najmaei S.; Liu Z.; Shi Y.; Kong J.; Lou J.; Ajayan P. M.; Yakobson B. I.; Idrobo J.-C. Intrinsic Structural Defects in Monolayer Molybdenum Disulfide. Nano Lett. 2013, 13, 2615–2622. 10.1021/nl4007479. 23659662

[ref25] Li Y.; Ma Y.; Zhao M.; Sun Q.; Dai Y.; Liu J.; Li H.; Dai X. The magnetism of intrinsic structural defects in monolayer MoTe_2_. J. Alloys Compd. 2018, 735, 2363–2372. 10.1016/j.jallcom.2017.12.041.

[ref26] Liu J.; Wang Y.; Xiao X.; Zhang K.; Guo N.; Jia Y.; Zhou S.; Wu Y.; Li Q.; Xiao L. Conversion of Multi-layered MoTe_2_ Transistor Between P-type and N-type and Their Use in Inverter. Nanoscale Res. Lett. 2018, 13, 291 10.1186/s11671-018-2721-0. 30242523 PMC6150881

[ref27] Qu D.; Liu X.; Huang M.; Lee C.; Ahmed F.; Kim H.; Ruoff R. S.; Hone J.; Yoo W. J. Carrier-Type Modulation and Mobility Improvement of Thin MoTe_2_. Adv. Mater. 2017, 29, 1606433 10.1002/adma.201606433. 28845903

[ref28] Guo Y.; Liu D.; Robertson J. Chalcogen vacancies in monolayer transition metal dichalcogenides and Fermi level pinning at contacts. Appl. Phys. Lett. 2015, 106, 173106 10.1063/1.4919524.

[ref29] Di Bartolomeo A.; Genovese L.; Giubileo F.; Iemmo L.; Luongo G.; Foller T.; Schleberger M. Hysteresis in the transfer characteristics of MoS2 transistors. 2D Mater. 2018, 5, 015014 10.1088/2053-1583/aa91a7.

[ref30] Zhu H.; Wang Q.; Cheng L.; Addou R.; Kim J.; Kim M. J.; Wallace R. M. Defects and Surface Structural Stability of MoTe_2_ under Vacuum Annealing. ACS Nano 2017, 11, 11005–11014. 10.1021/acsnano.7b04984. 29116754

[ref31] Seo S.-Y.; Moon G.; Okello O. F. N.; Park M. Y.; Han C.; Cha S.; Choi H.; Yeom H. W.; Choi S.-Y.; Park J.; Jo M.-H. Reconfigurable photo-induced doping of two-dimensional van der Waals semiconductors using different photon energies. Nat. Electron. 2021, 4, 38–44. 10.1038/s41928-020-00512-6.

[ref32] Wu E.; Xie Y.; Zhang J.; Zhang H.; Hu X.; Liu J.; Zhou C.; Zhang D. Dynamically controllable polarity modulation of MoTe_2_ field-effect transistors through ultraviolet light and electrostatic activation. Sci. Adv. 2019, 5, eaav3430 10.1126/sciadv.aav3430. 31058220 PMC6499594

[ref33] Rose H. Electron microscopy. Optik 1974, 39, 416–436.

[ref34] Okunishi E.; Ishikawa I.; Sawada H.; Hosokawa F.; Hori M.; Kondo Y. Visualization of Light Elements at Ultrahigh Resolution by STEM Annular Bright Field Microscopy. Microsc. Microanal. 2009, 15, 164–165. 10.1017/S1431927609093891.

[ref35] Liu X.; Islam A.; Guo J.; Feng P. X.-L. Controlling Polarity of MoTe_2_ Transistors for Monolithic Complementary Logic *via* Schottky Contact Engineering. ACS Nano 2020, 14, 1457–1467. 10.1021/acsnano.9b05502. 31909988

[ref36] Liu X.; Qu D.; Wang L.; Huang M.; Yuan Y.; Chen P.; Qu Y.; Sun J.; Yoo W. J. Charge Density Depinning in Defective MoTe_2_ Transistor by Oxygen Intercalation. Adv. Funct. Mater. 2020, 30, 2004880 10.1002/adfm.202004880.

[ref37] Okello O. F. N.; Yang D.-H.; Chu Y.-S.; Yang S.; Choi S.-Y. Atomic-level defect modulation and characterization methods in 2D materials. APL Mater. 2021, 9, 100902 10.1063/5.0062633.

[ref38] Fan H. B.; Yuen M. M. Material properties of the cross-linked epoxy resin compound predicted by molecular dynamics simulation. Polymer 2007, 48, 2174–2178. 10.1016/j.polymer.2007.02.007.

[ref39] Momma K.; Izumi F. *VESTA 3* for three-dimensional visualization of crystal, volumetric and morphology data. J. Appl. Crystallogr. 2011, 44, 1272–1276. 10.1107/S0021889811038970.

[ref40] Barthel J. Dr. Probe: A software for high-resolution STEM image simulation. Ultramicroscopy 2018, 193, 1–11. 10.1016/j.ultramic.2018.06.003. 29906518

