# Supplementary material for: Atomistic Probing of Defect-Engineered 2H-MoTe2 Monolayers
Source: ACS Nano. 2024 Feb 20;18(9):6927–35. doi: 10.1021/acsnano.3c08606 (PMC10919086; doi:10.1021/acsnano.3c08606)
Supplement: Supplementary file 1 — nn3c08606_si_001.pdf [file nn3c08606_si_001.pdf]

Supporting Information

Atomistic Probing of Defect-Engineered 2H-$MoTe_2$ Monolayers

Odongo Francis Ngome Okello[1,2,†], *Dong-Hwan Yang*[1,3,†], *Seung-Young Seo*[1], *Jewook Park*[1,3], *Gunho Moon*[1,3], *Dongwon Shin*[4], *Yu-Seong Chu*[5], *Sejung Yang*[6,7], *Teruyasu Mizoguchi*[8], *Moon-Ho Jo*[1,3,*], *and Si-Young Choi*[1,3,9,*]

[1] Department of Materials Science and Engineering, Pohang University of Science and Technology (POSTECH), 77 Cheongam-ro, Nam-gu, Pohang-si 37673, Republic of Korea.

[2] Samsung Electronics, Foundry Analysis & Engineering Team, Global Manufacturing & Infra Technology, Samsungjeonja-ro 1, Hwaseong-si 18448, Republic of Korea.

[3] Center for Van der Waals Quantum Solids, Institute of Basic Science (IBS), 77 Cheongam-ro, Nam-gu, Pohang-si 37673, Republic of Korea.

[4] Materials Science and Technology Division, Oak Ridge National Laboratory (ORNL), TN 37831, USA.

[5] Division of Biomedical Engineering, College of Health Sciences, Yonsei University, 1, Yeonsedae-gil, Heungeop-myeon, Wonju-si 26493, Republic of Korea.

[6] Department of Precision Medicine, Yonsei University, Wonju College of Medicine, 20 Ilsan-ro, Wonju-si 26426, Republic of Korea.

[7] Department of Medical Informatics and Biostatistics, Graduate School, Yonsei University, 20 Ilsan-ro, Wonju-si 26426, Republic of Korea.

[8] Institute of Industrial Science, The University of Tokyo, Komaba, Meguro 4-6-1, Tokyo 153-8505, Japan.

[9] Department of Semiconductor Engineering, POSTECH, 77 Cheongam-ro, Nam-gu, Pohang-si 37673, Republic of Korea.

* Correspondence : (M.-H. J.) mhjo@postech.ac.kr; (S.-Y. C.) youngchoi@postech.ac.kr

† O. F. N. O. and D.-H. Y. are equally contributed.

Supporting Text 1. Experimental and simulation HAADF-STEM analyses of defects in 200˚C-vacuum-annealed 2H-$MoTe_2$ ML.

To verify the defect species visualized in the 200˚C-vacuum-annealed 2H-$MoTe_2$ (VA 2H-$MoTe_2$) ML, we performed high-resolution HAADF-STEM simulation analysis and compared the intensity profiles with the experimental results. The HAADF-STEM images were simulated from atomic models constructed taking into consideration the possible prevalent defects. **Figure S1** shows **a** experimental HAADF-STEM image of Te_{ad1}, **b** (**c**) and simulated HAADF-STEM images of Te_{ad1} (Mo_{ad1}). The intensity profiles shown in **d** are extracted from **a-b** light-green solid, and **c** light-gray solid diagonal rectangles, respectively. The corresponding atomic models are presented at the bottom for clarity. Based on intensity profiles, the experimental result (open light-green squares) is comparable with that of Te_{ad1} (light-green solid line).

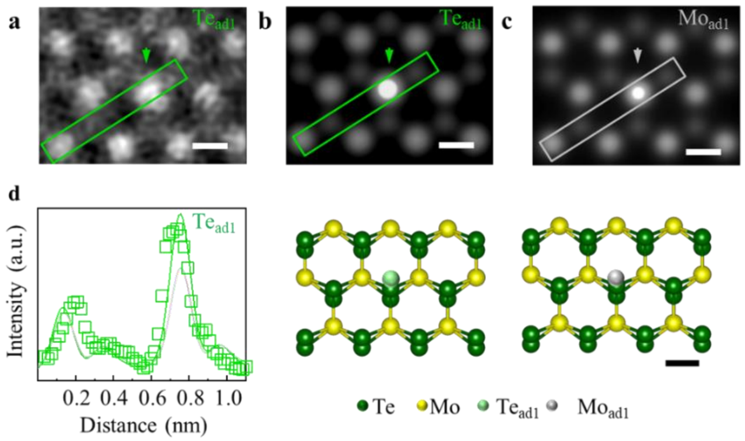

Figure S1. Inspection of Te_{ad1} defect. **a** Experimental HAADF-STEM image of Te_{ad1} observed in VA 2H-$MoTe_2$ ML. **b-c** (Top panels) Simulated HAADF-STEM image of Te_{ad1} and Mo_{ad1} respectively. The defect sites are indicated by the colored arrows. (Bottom panels) Corresponding atomic models as top panels. Scale bars; 0.2 nm. **d** Experimental (open light-green squares) and simulated (light-green/light-gray line) HAADF-STEM intensity profiles extracted from **a-c** along the diagonal rectangles. We confirm the defect observed in the experimental HAADF-STEM image as Te_{ad1}. The color-codes are the same as in Figure 2, where Mo-related defects are marked in light-gray for comparison.

Figure S2 shows **a** experimental HAADF-STEM image of Te_{ad2}, and **b** (**c**) simulated HAADF-STEM images of Te_{ad2} (Mo_{ad2}). The intensity profiles shown in **d** are extracted from **a-b** orange solid, and **c** light-gray solid diagonal rectangles, respectively. The corresponding atomic models are presented at the bottom for clarity. Based on intensity profiles, the experimental intensity (open orange squares) profile is comparable with that of Te_{ad2} (orange solid line).

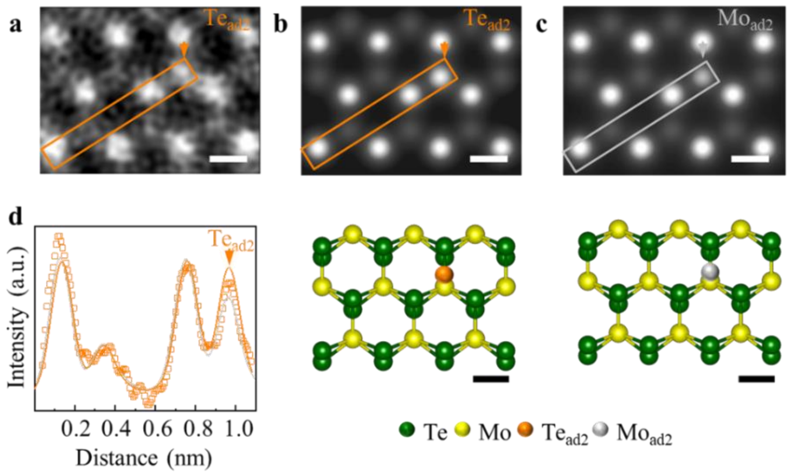

Figure S2. Inspection of Te_{ad2} defect. **a** Experimental HAADF-STEM image of Te_{ad2} observed in VA 2H-$MoTe_2$ ML. **b-c** (Top panels) Simulated HAADF-STEM image of Te_{ad2} and Mo adatom on Mo; Mo_{ad2}, respectively. The defect sites are indicated by the colored arrows. (Bottom panels) Corresponding atomic models of the simulated HAADF-STEM images in the top panels. Scale bars; 0.2 nm. **d** Experimental (open orange squares) and simulated (solid orange/light-gray lines) HAADF-STEM intensity profiles extracted from **a-c** along the diagonal rectangles. We confirm the defect observed in the experimental HAADF-STEM image as Te_{ad2}. The color-codes are the same as in Figure 2, where Mo-related defects are marked in light-gray for comparison.

Supporting Text 2. Experimental and simulation HAADF-(ABF-)STEM analysis of defects in 532-nm-laser-illuminated 2H-$MoTe_2$ ML.

To verify the defect species observed in the 532-nm-laser-illuminated 2H-$MoTe_2$ (LI 2H-$MoTe_2$) ML, we performed high-resolution HAADF-(ABF-)STEM simulation analysis and compared the intensity profiles with the experimental results. The HAADF-(ABF-)STEM images were simulated from atomic models constructed taking into consideration the possible prevalent defects.

Figure S3 shows **a** experimental HAADF-STEM image of V_{Mo} & Mo_{int}, and **b** (**c**) simulated HAADF-STEM images of V_{Mo} & Mo_{int} (V_{Mo} & Te_{int}). The intensity profiles shown in **d** are extracted from **a** and **c** along the purple/solid, **b** dark yellow solid diagonal rectangles, respectively. The corresponding atomic models are presented at the bottom for clarity. Based on intensity profiles, the experimental intensity (open purple squares) profile is comparable with that of V_{Mo} & Mo_{int} (purple solid line).

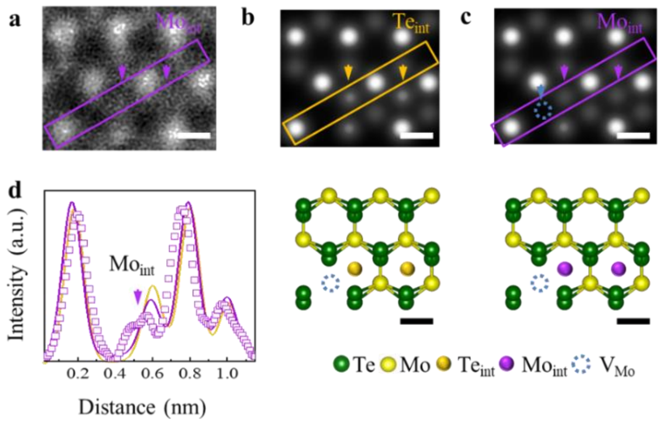

Figure S3. Inspection of V_{Mo} & Mo_{int}. **a** Experimental HAADF-STEM image of V_{Mo} & Mo_{int} pair observed in LI 2H-$MoTe_2$ ML. **b-c** (Top panels) Simulated HAADF-STEM image of V_{Mo} & Mo_{int}. The defect sites are indicated by the colored arrows. (Bottom panels) Corresponding atomic models as top panels. Scale bars; 0.2 nm. **d** Experimental (open purple squares) and simulated (solid dark yellow/purple) HAADF-STEM intensity profiles extracted from **a-c** along the diagonal rectangles. We confirm the defect observed in the experimental HAADF-STEM image as V_{Mo} & Mo_{int} pair. The color-codes are the same as in Figure 2, where Te_{int} are marked in dark yellow for comparison.

Figure S4 illustrates the experimental set-up to optimize the 532-nm-laser illumination set-up. With the focused the laser beam, the bulk sample prepared by Focus Ion Beam (FIB) was damaged as illustrated in Figure S4**a**. Here, the distance between laser and sample was 40 cm without ND filter. To reduce the sample damage from focused laser beam, we placed an ND filter between the laser and the sample with a distance of 30 cm (d_1), and adjusted the distances between the ND filter and the TEM sample of 30 cm (d_2).

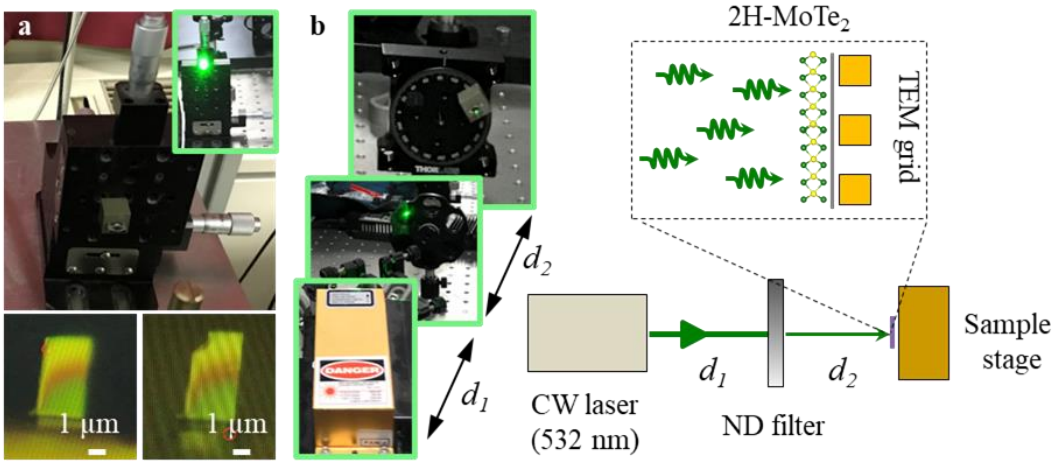

Figure S4. a (Top) Experimental set-up for laser-illumination before optimization to illuminate the TEM sample. (Bottom-left) ((Bottom-right)) Cross-sectional FIB sample of bulk 2H-$MoTe_2$ before (after) illuminated with focused laser. Even for the bulk 2H-$MoTe_2$, sample was hugely damaged. **b** Optimized laser-illumination set up for TEM sample by inserting ND filter with adjusting distance between laser source-ND filter (d_1) and ND filter-TEM sample (d_2). The distance changes result in defocus to the TEM sample, which suggests the mild laser-illumination condition to monolayer 2H-$MoTe_2$ sample. On the right is a schematic illustration of the experimental set-up.

To inspect oxygen-related defect in LI 2H-$MoTe_2$ ML, we constructed and performed several atomic simulations for 2H-$MoTe_2$ MLs taking into consideration potential atomic defects (**Figure S5**). Concurrently, we incorporated ABF-STEM simulation approach that is highly sensitive for low Z-element (Z = 8) such as oxygen defects present at the Te vacancy site. The HAADF-STEM image is expected to

show minimal contrast at the Te vacancy defect sites. For a V_{Te1+1O}, we could hardly detect oxygen contrast in the HAADF-STEM simulation image, due to the present of one Te atom at the same atomic column as oxygen atomic defect. However, a close-up look at the ABF-STEM reveal slightly enhanced contrast as indicated by red arrow in Figure S5**c**. For V_{Te2+1O} and V_{Te2+2O}, the ABF-STEM simulation clearly revealed enhanced oxygen contrast, since the two Te atoms are missing at the V_{Te2} defect site (Figure S5**e-f**).

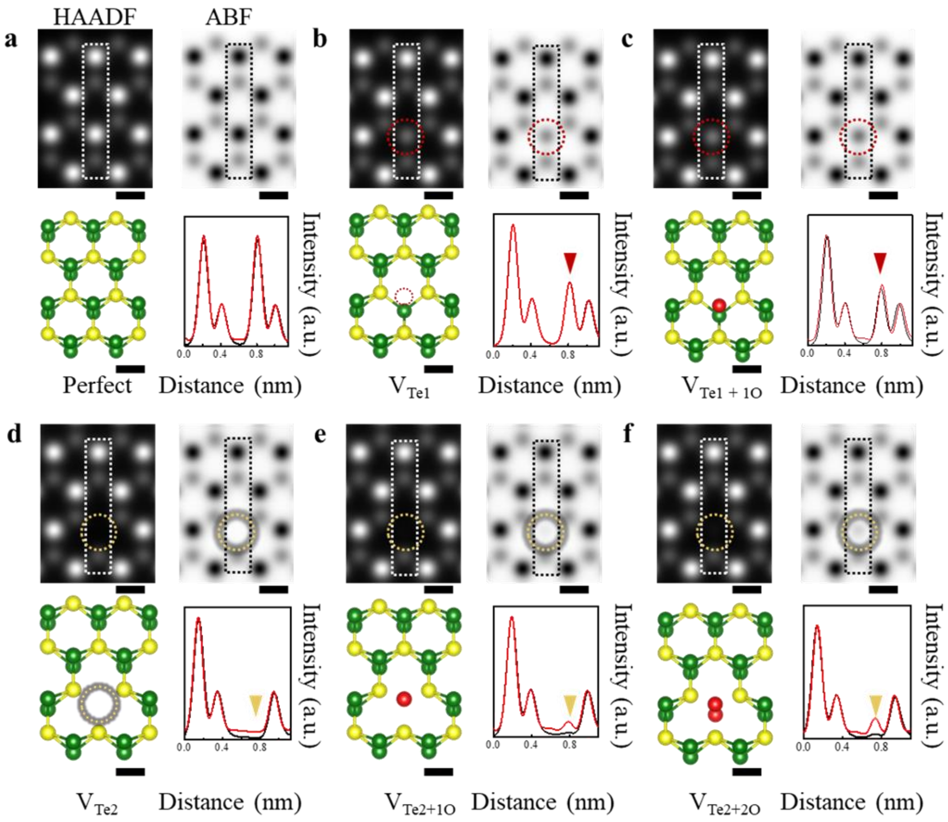

Figure S5. Inspection of oxygen-related defects. **a-f** (Top-left)-(Top-right) Simulated HAADF-(grey scale) and ABF-(false color) STEM analysis of **a** Perfect (control group), **b** V_{Te1}, **c** V_{Te1+1O}, **d** V_{Te2}, **e** V_{Te2+1O}, and **f** V_{Te2+2O}, respectively. (Bottom-left)-(Bottom-right) Corresponding atomic models and intensity profile as top panels. From all intensity profiling, oxygen contrast is clearly detectable in the ABF-STEM images of V_{Te2+1O} and V_{Te2+2O}. The V_{Te1+1O} shows negligible oxygen contrast compared to V_{Te1} and V_{Te1+1O} due to the presence of Te atom in the same column as oxygen-related defect. Scale bars; 0.2 nm.

Figures S6a-b show experimental HAADF- and ABF-STEM image (top panels) and simulated HAADF- and ABF-STEM simulation (middle panels) of V_{Te1+1O}. The extracted intensity profiles from **a** and **b** along the red dotted rectangles are shown below the HAADF- and ABF-STEM simulation images. The corresponding atomic models are presented at the bottom of **b** for clarity. Based on intensity profiles, a weak contrast-enhancement was observed in the ABF-STEM as indicated by the red arrow. Figures S6**c-d** show experimental HAADF- and ABF-STEM image (top panels) and simulated HAADF- and ABF-STEM simulation (middle panels) of V_{Te2+2O}. The extracted intensity profiles from **c** and **d** along the yellow dotted rectangles are shown below the HAADF- and ABF-STEM simulation images. The corresponding atomic models are presented at the bottom of the intensity profile for clarity. By closely observing the intensity profiles, we can see a strong contrast at the defect site in ABF-STEM intensity profile (indicated by the yellow arrow) which is attributed to oxygen defect. The contrast is more enhanced in the column with two missing Te atoms (V_{Te2}).

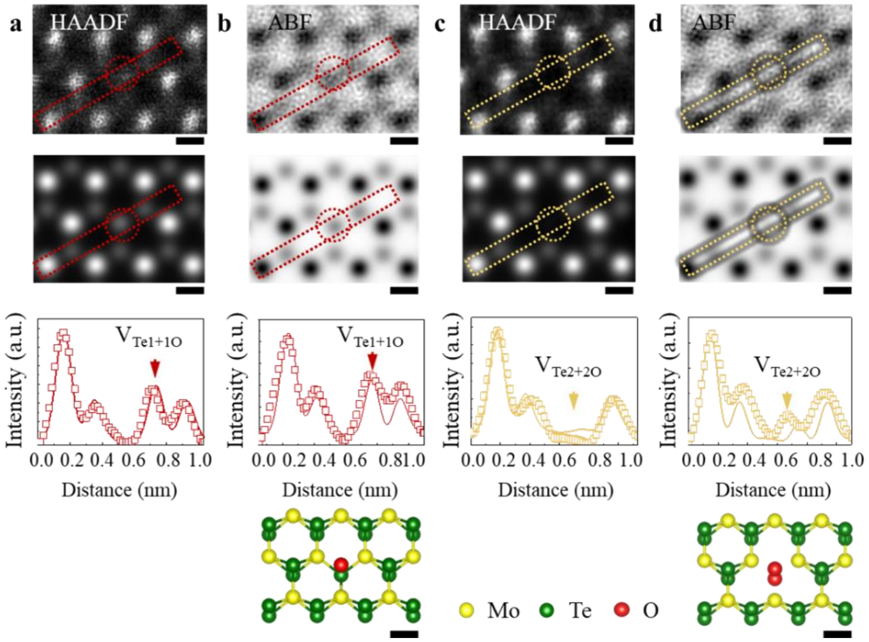

Figure S6. a-b (Top panels) ((Middle panels)) Experimental (simulated) HAADF- and ABF-STEM images of LI 2H-$MoTe_2$ ML, with V_{Te1+1O} indicated by red dotted circles. Experimental (open red squares) and simulated (solid red lines) HAADF-(ABF-)STEM intensity profiles extracted from **a-b** along the diagonal rectangles are shown below the simulated images. **c-d** (Top panels) ((Middle panels)) Experimental (simulated) HAADF- and ABF-STEM images of LI 2H-$MoTe_2$ ML with V_{Te2+2O} indicated by yellow dotted circles. Experimental (open yellow squares) and simulated (solid yellow lines) HAADF-(ABF-)STEM intensity profiles extracted from **c-d** along the diagonal rectangles are shown at the bottom. The oxygen contrast and intensity ratio for V_{Te1+1O} is diminished. However, the oxygen contrast is well pronounced at the V_{Te2+2O} site. For clarity, the atomic models embedded with V_{Te1+1O} and V_{Te2+2O} are presented at the bottom of ABF-STEM intensity profiles. Scale bars; 0.2 nm.

Supporting Text 3. A comparison between ABF-STEM image analyses of 200℃-vacuum-annealed and 532-nm-laser-illuminated 2H-$MoTe_2$ MLs.

To elucidate the presence of oxygen-related defect in LI 2H-$MoTe_2$, we analyzed and compared the experimental ABF-STEM images for both VA 2H-$MoTe_2$ and LI $MoTe_2$ MLs. The top panels in **Figures S7a-b** are the ABF-STEM images with V_{Te2} and V_{Te2+2O} for VA 2H-$MoTe_2$ and LI 2H-$MoTe_2$, respectively. Figure S7**c** profiles the corresponding ABF-STEM intensities extracted from Figure S7**a-b** along the regions marked by yellow dotted rectangles. Remarkably, the ABF-STEM intensity profile for LI 2H-$MoTe_2$ (filled yellow squares) reveal enhanced intensity (yellow arrow) at the defect site which is not observable in the VA 2H-$MoTe_2$ (open yellow squares). This further confirms that oxygen defect is only achievable by laser-illumination.

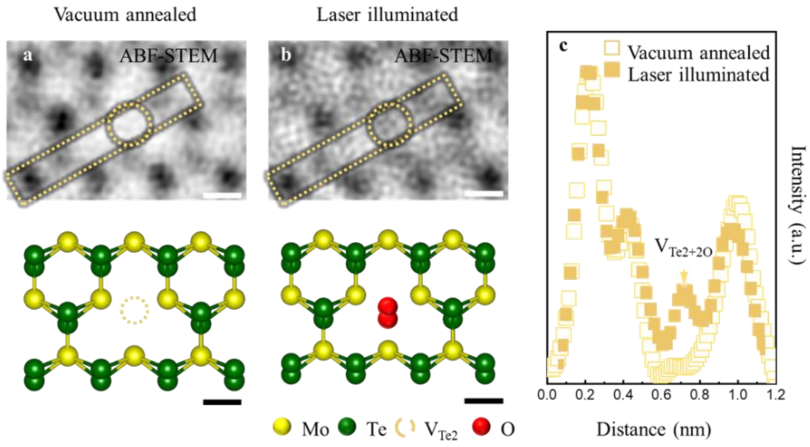

Figure S7. a-b (Top panels) ABF-STEM intensity profiles for VA 2H-$MoTe_2$ ML with V_{Te2} and LI 2H-$MoTe_2$ ML with V_{Te2+2O}, respectively. The corresponding atomic models with the same color-codes as Figures 2-3 are presented at the bottom. Scale bars; 0.2 nm. **c** ABF-intensity profile of VA 2H-$MoTe_2$ (open yellow squares) and LI 2H-$MoTe_2$ (filled yellow squares). The weak contrast attributed to chemisorbed oxygen at the Te vacancy site (filled yellow arrow) is only evident in LI 2H-$MoTe_2$.

Supporting Text 4. Experimental HAADF/ABF-STEM analysis of oxygen plasma-treated 2H-$MoTe_2$ ML.

To directly verify the presence of oxygen atoms at the Te vacancy-site, we also analyzed HAADF- and ABF-STEM images of 2H-$MoTe_2$ MLs exposed to oxygen plasma. Previous studies have demonstrated that exposing 2H-$MoTe_2$ flakes to oxygen-rich conditions, *e.g.*, oxygen plasma, results in the creation of Te vacancies with subsequent adsorption/chemisorption of oxygen at these sites resulting in p-type characteristics[1-3]. As expected, we could identify a weak atomic contrast at the V_{Te2} site, consistent with our finding in LI 2H-$MoTe_2$ ML.

Figure S8a depicts HAADF-STEM image of the oxygen plasma-treated 2H-$MoTe_2$ (PT 2H-$MoTe_2$). Here, The representative atomic defects are shown by dotted rectangles marked (i)-(v), respectively. Figures S8**b-d** show enlarged HAADF-STEM micrograph from the dotted rectangle (i)-(v) in Figure S8**a**. The representative defects in Figure S8**b-d** were identified as (i) Te_{ad1} (light-green solid circle), (ii) Te_{ad2} (orange solid circle) and (iii) Te_{int} (dark-yellow solid circle), respectively. The corresponding atomic model is also shown on the right side where Te_{ad1}, Te_{ad2}, and Te_{int} defects are denoted by the light-green, orange, and dark-yellow spheres, respectively. Figures S8**e-f** are enlarged HAADF- (top) and ABF- (middle) STEM images extracted from the dotted rectangles (vi)-(v) in Figure S8**a**. The corresponding atomic models are presented at the bottom for clarity. Figure S8**g** profiles the corresponding HAADF- and ABF-STEM intensities extracted from Figure S8**f** along the regions marked by dotted yellow rectangles. As expected, the ABF-STEM intensity profile (open yellow squares) illustrates presence of a weak contrast at the V_{Te2} site which is absent in the HAADF-STEM intensity profile (open gray squares). This finding further ascertain the presence of the oxygen atoms adsorbed/chemisorbed at V_{Te2} and is in good agreement with our result obtained using LI 2H-$MoTe_2$.

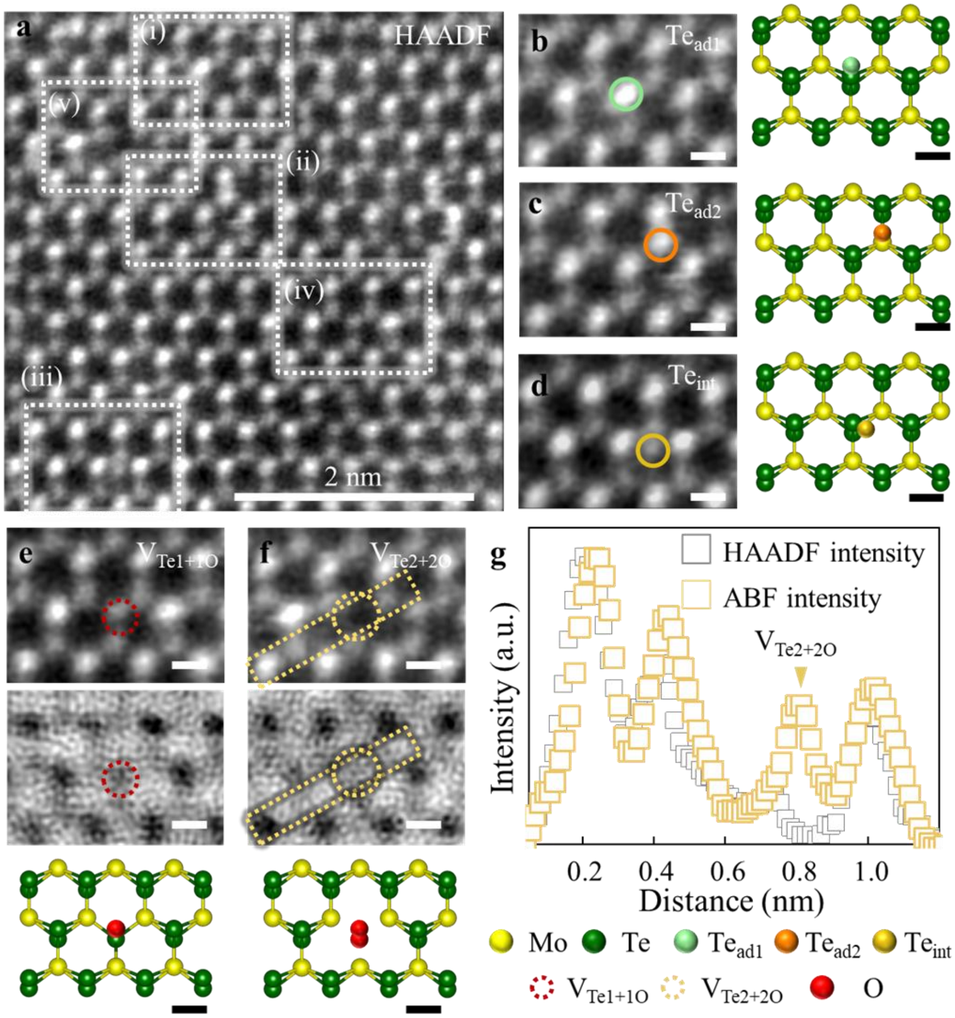

Figure S8. Point defect type exploration for oxygen plasma-treated (PT) 2H-$MoTe_2$ ML. **a** Wide-view experimental HAADF-STEM of PT 2H-$MoTe_2$ ML. Typical defects denoted by dotted rectangle (i)-(v) in **a** represent (i) Te_{ad1}, (ii) Te_{ad2}, (iii) Te_{int}, (vi) V_{Te1+1O}, and (v) V_{Te2+2O}, respectively. **b-d** (Left panels) Enlarged HAADF-STEM micrographs in **a** revealing (i) Te_{ad1} (green dotted circle), (ii) Te_{ad2} (orange dotted circle), and (iii) Te_{int} (dark yellow), respectively. (Right panels) Corresponding atomic model in left panels. **e-f** (Top panels) Enlarged HAADF-STEM micrographs in **a** revealing (vi) V_{Te1+1O} (dotted red circle) and (v) V_{Te2+2O} (dotted yellow circle), respectively. (Middle panels) Corresponding ABF-STEM as top panels illustrating enhanced atomic contrast attributed to oxygen adsorption/chemisorption at each V_{Te1} and V_{Te2} sites. (Bottom panels) Corresponding atomic configurations with oxygen atomic defects (red spheres). Scale bars; 0.2 nm. **e** Intensity profiles for HAADF (open gray squares) and ABF (open yellow squares) region extracted along the dotted diagonal yellow rectangles in top and bottom panels in **d**, respectively. Note that oxygen contrast is detectable only in ABF-STEM (yellow arrow) profile.

Supporting Text 5. Deep learning-based inspection of point defects in pristine and defect-engineered 2H-$MoTe_2$ MLs.

Figure S9 illustrates the point defect classification workflow by deep learning models. In the deep learning (DL)-processing, the input images are fed to three deep learning models; (i) unit cell detection, (ii) Te on-site defect (Te defect) and (iii) Mo on-site defect (Mo defect) classification as mentioned in the Methods. From the defect-classified results of Te defect and Mo defect, each image is fed to Faster R-CNN (Regions with Convolutional Neural Networks to crop unit cells and location of unit cells in the post-processing. The combination of Te defect and Mo defect classification results from each unit cell finally determines defect types *e.g.* V_{Te1} from Te defect and V_{Mo} from Mo defect resulting in V_{Te1}+V_{Mo}.

Figure S10a presents the architecture of FCN to classify Te defect as an example. The configuration of FCN is divided into an “encoding” module and a “decoding” module, respectively. The encoding module consists of maxpooling layer, convolution layer, batch normalization layer, and PReLU activation function. The decoding module is changed to a transposed convolution layer instead of a maxpooling layer in the encoding module. Feature concatenation is used to combine feature maps in the encoding module in the same stage with the decoding module. The point defect types are classified through encoding and decoding process. Either Te defect or Mo defect can be identified. Figures S10**b-c** presents the train/validation loss curves during FCN training for Te defect and Mo defect types. Note that the train/validation loss values are very low even at the early stage of training, which means learning process fits well to the point defect analysis. The left panels of Figures S11**d-e** are input HAADF-STEM images with randomly distributed point defects in crystal matrices. Compared to the ground truth of input images (middle panels), the trained-FCN models identify the defect types in either Te on-site or Mo on-site defects, as illustrated in the right panels in Figure S10 and as mentioned in Figure S9.

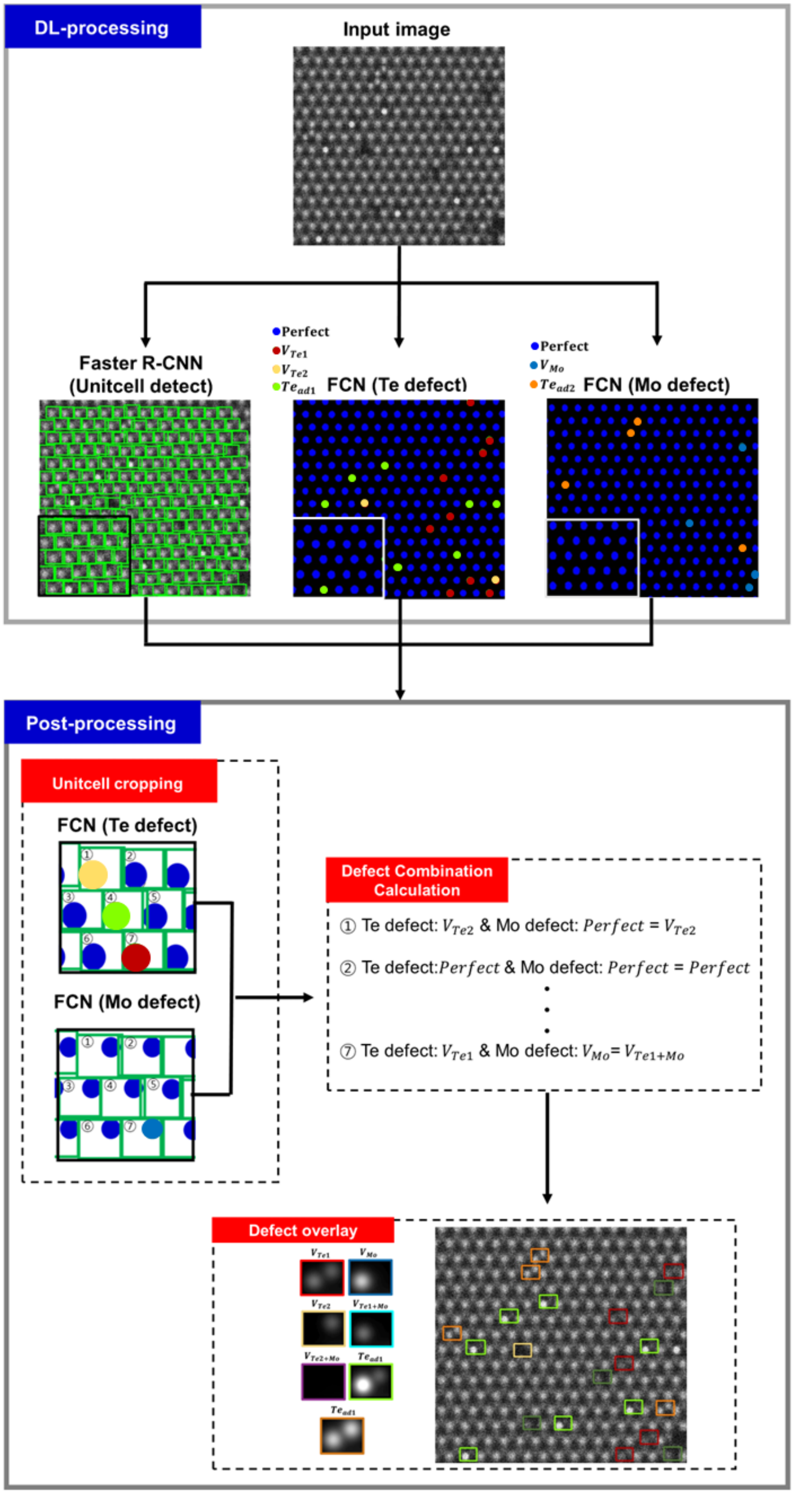

Figure S9. Schematic representation of deep learning method. Deep learning (DL) processing: Input STEM images are fed to each of the three deep learning models. Post-processing: Point defect classification results of Te defect and Mo defect, each image is fed to Faster R-CNN to detect unit cell and unit cell locations. With the combination of Te defect and Mo defect for each unit cell area, the final point defect types are determined. Finally, the classification results corresponding to each types are overlaid on the input STEM images.

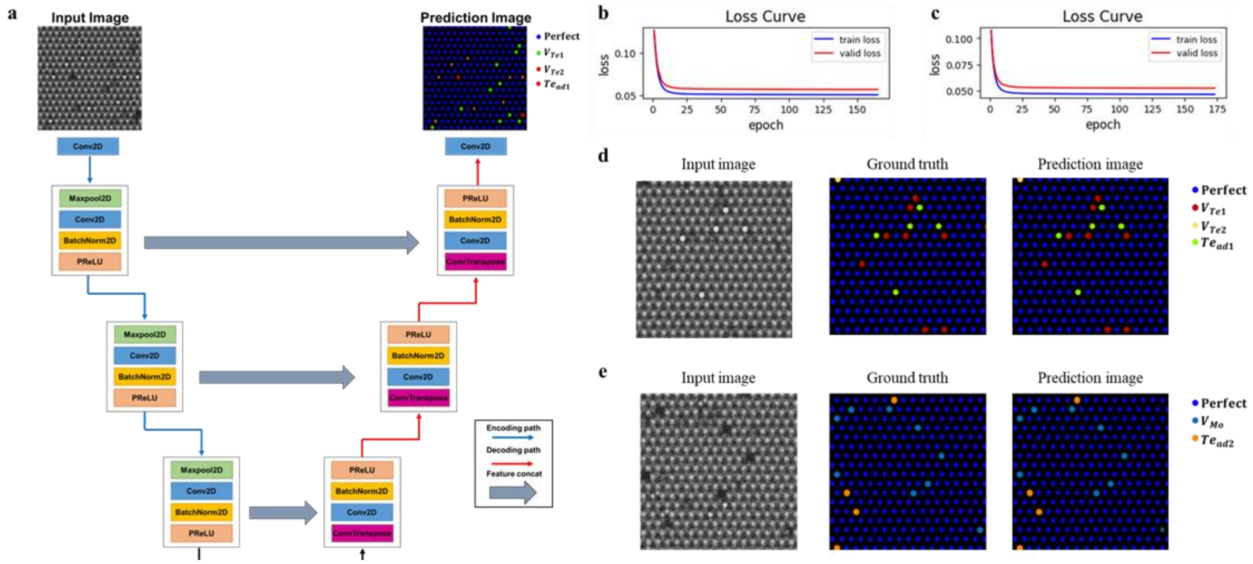

Figure S10. a Fully Convolution Network (FCN) that segments defect types in the Te on-site. The configuration of the network is divided into an “encoding” module and a “decoding” module, respectively. The encoding module consists of maxpooling layer, convolution layer, batch normalization layer, and PReLU (Parametric Rectified Linear Unit) activation function. The decoding module is changed to a transposed convolution layer instead of a maxpooling layer in the encoding module. Feature concatenation is used to combine feature maps in the encoding module in the same stage with the decoding module. **b-c** The loss curves for train/validation for Te on-site and Mo on-site defect classifications by FCN model. **d-e** (Left panel) Input HAADF-STEM images for defect examination with randomly distributed point defects. (Middle panel) Ground truth of left panels. (Right panel) The predicted point defect types by trained FCN models. The color-codes are the same as Figures 2-3, except the Perfect type (blue). Scale bars; 0.5 nm.

Figure S11 depicts application of automatic point defect classification algorithm to the experimental HAADF-STEM image of pristine 2H-$MoTe_2$ as a basic point defect examination. The top and bottom panels in Figure S11**a** illustrates estimated point defects and corresponding ground truth (labelled by human), respectively. In Figure S11**b**, we present the overall summary of species and concentration of point defects; V_{Te1} (red) with concentration of $0.58\times10^{14}/cm^2$ (total analyzed area of $1.38\times10^{15}/cm^2$ (13.8 nm^2). Figure S11**c** is a confusion matrix of point defect classification performances; the defect classification accuracies for each defect species were 100.0% (Perfect), 100.0% (V_{Te1}) of total 202 classifications. Figure

S11**d**, shows the corresponding device characterization (back gated-FET) of pristine 2H-$MoTe_2$. As discussed in Figure 1, the Te-vacancies manifest n-type character to 2H-$MoTe_2$, which was defined by our deep learning models.

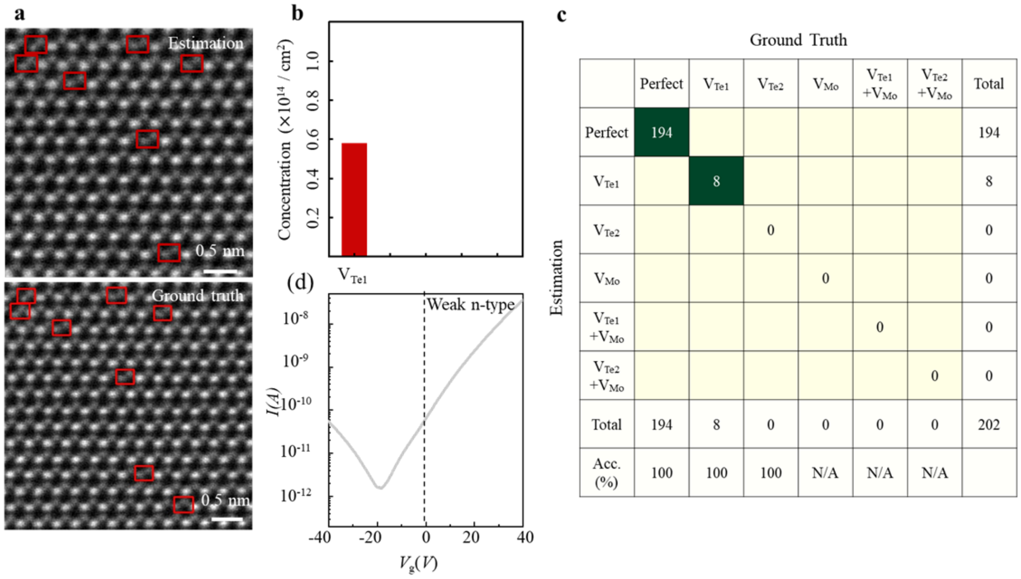

Figure S11. a (Top) Estimated point defect classification results of pristine 2H-$MoTe_2$ ML. The color-codes are the same as in Figure 2. (Bottom) Corresponding ground truth of top panel. **b** (Top) Defect concentration in pristine 2H-$MoTe_2$ revealed by deep learning. **c** Confusion matrices for point defect estimation performance of total 202 input unit cells of pristine ML 2H-$MoTe_2$. **d** (Bottom) Transfer (I-V_g) plot for pristine 2H-$MoTe_2$ ML exhibiting weak n-type character by V_{Te1} as revealed by **a**.

Figures S12 presents the additional statistical point defect classification results of VA 2H-$MoTe_2$. The top and bottom panels in Figures S12**a-b** illustrate the estimated point defects and corresponding ground truth (labelled by human), respectively. The orange arrows in top panel are misclassified to Perfect; ground truth of Te_{ad2}. All the color-codes for defect species are the same as Figure 2. Figure S12**c** is a confusion matrix of point defect classification performances; the defect classification accuracies for each defect species were 100.0% (Perfect), 100.0% (V_{Te1}), 100.0% (V_{Te2}), 80.0% (Te_{ad1}), and 63.6% (Te_{ad2}) of total 297 classifications. Figure S12**d** corresponds to device characterization (back gated-FET) of VA 2H-

MoTe$_2$, exhibiting strong n-type character (black) compared to the weak n-type of pristine 2H-MoTe$_2$ (light-gray).

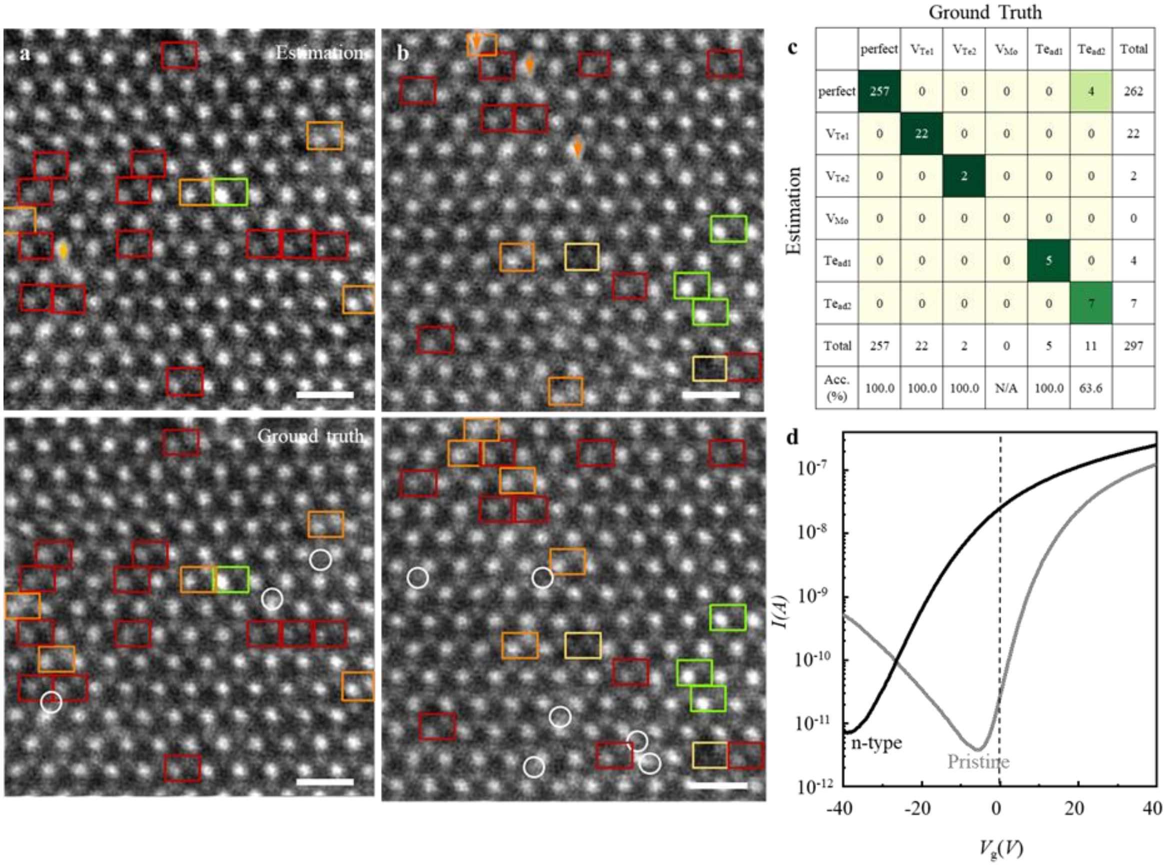

Figure S12. Point defect classification performance evaluation of VA 2H-MoTe$_2$ MLs. **a-b** (Top panel) Estimated point defect classification results by deep learning for VA 2H-MoTe$_2$ ML. (Bottom panel) Corresponding ground truth of top panel. The orange arrows in **a** and **b** are misclassified to Perfect; ground truth of Te_{ad1}. Note that Perfect ones are not indicated though they were classified by deep learning. Color-codes are all the same as Figures 2-3 for defect types. Scale bars; 0.5 nm. The interstitial defects are denoted by white circles. **c** Confusion matrices for point defect estimation performance of total 297 input unit cells of vacuum-annealed ML 2H-MoTe$_2$. **d** Transfer (I-V_g) plot for VA 2H-MoTe$_2$ ML exhibiting strong n-type character (black) compared to pristine 2H-MoTe$_2$ (weak n-type, grey) by defined defect types.

Figures S13 presents additional statistical point defect classification results of LI 2H-$MoTe_2$. The top and bottom panels in Figure S13**a-d** illustrate estimated point defects and ground truth (labelled by human), respectively. The blue arrow in top panel **d** is misclassified to Perfect; ground truth of V_{Mo}. All the color-codes for defect species are the same as Figure 3. Figure S13**e** is a confusion matrix of point defect classification performances; the defect classification accuracies for each defect species were 100.0% (Perfect), 100.0% (V_{Te1+1O}), 100.0% (V_{Te2+2O}), and 83.3% (V_{Mo}), and 63.6% (Te_{ad2}) of total 616 classifications. Figure S13**f** corresponds to device characterization (back gated-FET) of LI 2H-$MoTe_2$, exhibiting strong p-type character (green) compared to the weak n-type of pristine 2H-$MoTe_2$ (light-gray).

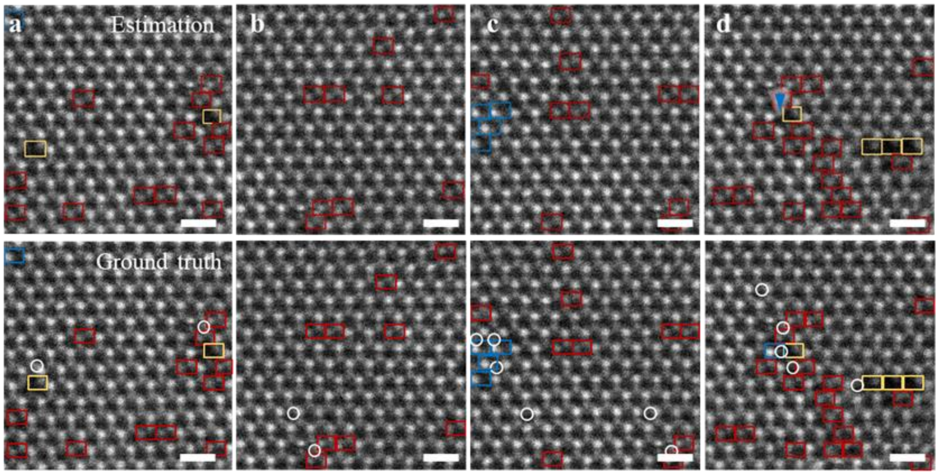

e Ground Truth (columns) / Estimation (rows)

	perfect	V_{Te1+1O}	V_{Te2+2O}	V_{Mo}	Te_{ad1}	Te_{ad2}	Total
perfect	555	0	0	1	0	0	556
V_{Te1+1O}	0	49	0	0	0	0	49
V_{Te2+2O}	0	0	6	0	0	0	6
V_{Mo}	0	0	0	5	0	0	5
Te_{ad1}	0	0	0	0	0	0	0
Te_{ad2}	0	0	0	0	0	0	0
Total	555	49	6	6	0	0	616
Acc. (%)	100.0	100.0	100.0	83.3	N/A	N/A	

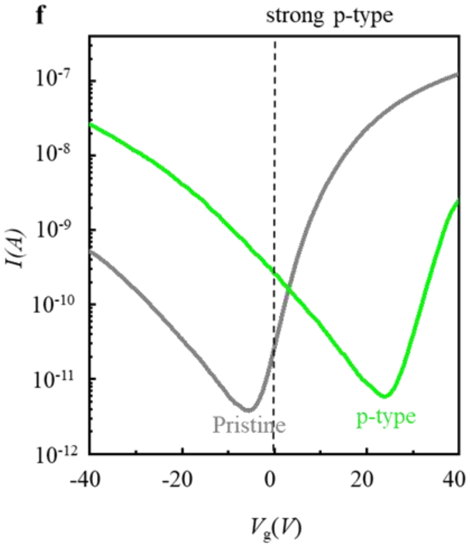

Figure S13. Point defect classification performance evaluation of LI 2H-$MoTe_2$ MLs. **a-d** (Top panels) Estimated point defect classification results by deep learning for LI 2H-$MoTe_2$ ML. (Bottom panels) Corresponding ground truth of top panels. The blue arrow in **d** is misclassified to Perfect; ground truth of V_{Mo}. Note that Perfect ones are not indicated though they were classified by deep learning. Color-codes are all the same as Figures 2-3 for defect types. Scale bars; 0.5 nm. The interstitial defects are denoted by white circles. **e** Confusion matrices for point defect estimation performance of total 616 input unit cells of LI 2H-$MoTe_2$ ML. **f** Transfer (I-V_g) plot for LI 2H-$MoTe_2$ ML exhibiting strong p-type character (green) compared to pristine 2H-$MoTe_2$ (weak n-type, gray) by defined defect types

Figures S14 presents point defect classification results of PT 2H-$MoTe_2$. The top and bottom

panels in Figure S14**a-c** illustrate estimated point defects and ground truth (labelled by human), respectively. The (i) orange, (ii) light-green, (iii) red arrows in top panels are all misclassified to Perfect; ground truth of (i) Te_{ad2}, (ii) Te_{ad1}, and (iii) V_{Te1+1O}, respectively. All the color-codes for defect species are the same as Figure 3. Figure S14**d** is a confusion matrix of point defect classification performances; the defect classification accuracies for each defect species were 100.0% (Perfect), 97.5% (V_{Te1+1O}), 100.0% (V_{Te2+2O}), 85.7% (Te_{ad1}), and 88.0% (Te_{ad2}) of total 477 classifications. In Figure S15**e**, we present the overall summary of species and distribution of point defects (left), and corresponding device characterization (back gated-FET) of PT 2H-$MoTe_2$, exhibiting strong p-type character (green). It is evident that oxygen plasma treatment generates V_{Te1+O} (blue), Te_{ad2} (light-blue), Te_{ad1} (sky-blue), V_{Te2+2O} (light-gray). The most dominant defect species is (i) V_{Te1+O}, followed by (ii) Te_{ad2}, (iii) Te_{ad1}, and (iv) V_{Te2+2O}, with the concentration being (i) $1.06\times10^{14}/cm^2$, (ii) $0.60\times10^{14}/cm^2$, (iii) $0.160\times10^{14}/cm^2$ and (iv) $0.14\times10^{14}/cm^2$ respectively (total analyzed area of $3.68\times10^{15}/cm^2$ (36.8 nm^2).

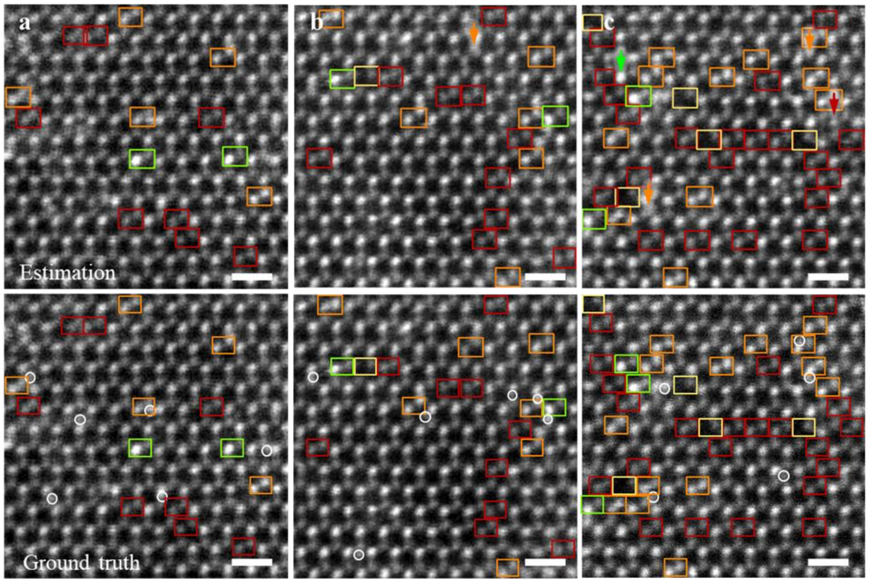

d Ground Truth (columns) / Estimation (rows)

	perfect	V_{Te1+1O}	V_{Te2+2O}	V_{Mo}	Te_{ad1}	Te_{ad2}	Total
perfect	400	1	0	1	1	3	405
V_{Te1+1O}	0	39	0	0	0	0	39
V_{Te2+2O}	0	0	5	0	0	0	5
V_{Mo}	0	0	0	0	0	0	0
Te_{ad1}	0	0	0	0	6	0	6
Te_{ad2}	0	0	0	0	0	22	22
Total	400	40	5	0	7	25	477
Acc. (%)	100.0	97.5	100.0	N/A	85.7	88.0	

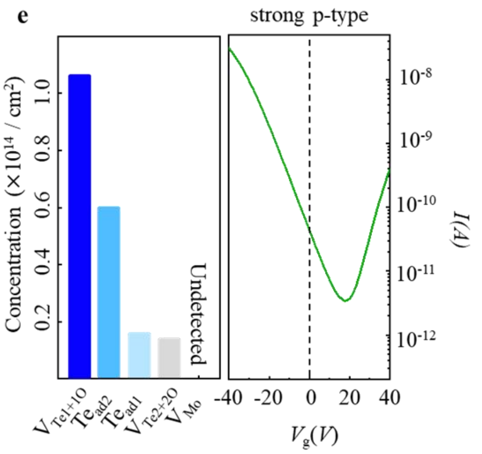

Figure S14. Point defect classification performance evaluation of PT 2H-$MoTe_2$ MLs. **a-c** (Top panels) Estimated point defect classification results by deep learning for PT 2H-$MoTe_2$ ML. (Bottom panels) Corresponding ground truth of top panels. The orange arrow in **b** is misclassified to Perfect; ground truth of Te_{ad2}. Similarly, the green, yellow, and red arrows in **c** are all misclassified to Perfect; ground truth of Te_{ad1}, V_{Te2+2O}, and V_{Te1+1O}, respectively. Note that Perfect regions are not indicated though they were classified by deep learning. Color-codes are all the same as Figures 2-3 for defect types. Scale bars; 0.5 nm. The interstitial defects are denoted by white circles. **d** Confusion matrices for point defect estimation performance of total 477 input unit cells of PT 2H-$MoTe_2$ ML. **e** (Left) Statistical point defects classification by deep learning in PT 2H-$MoTe_2$. V_{Te1+1O} (blue), Te_{ad2} (light-blue), Te_{ad1} (sky-blue), V_{Te2+2O} (light-gray). (Right) Transfer (I-V_g) plot for PT 2H-$MoTe_2$ ML exhibiting strong p-type character (green) by defined defect types.

For VA 2H-$MoTe_2$ and LI 2H-$MoTe_2$, the classification accuracies for Te_{ad2} and V_{Mo} were the lowest; 63.6% and 83.3%, respectively. For a close-up view of VA 2H-$MoTe_2$ (left and middle panel in **Figure S15a**), the orange arrow denotes the mis-classification to Perfect; the ground truth of Te_{ad2}. These trends are often observed as illustrated in confusion matrix (Figure S12). To address this confusion of FCN for Te_{ad2} *vs* Perfect type in VA 2H-$MoTe_2$, we profiled the intensities in HAADF-STEM of (i) simulated Te_{ad2} (orange open squares), (ii) simulated Perfect (gray open squares), and (iii) experimental Te_{ad2} (orange solid line), which extracted from white dotted diagonal rectangles (Figure S15**c**). By comparing the intensities, the contrast of "experimental Te_{ad2} site" locates between "simulated Te_{ad2}" and "simulated Perfect type", though it reaches to the simulated Te_{ad2}. These contrasts variation at Te_{ad2} may contribute the confusion for FCN between Te_{ad2} *vs* Perfect types. Also, the absolute number of Te_{ad2} observed in our experimental images *i.e.* 11 counts can contribute to the superficially-lower accuracies for Te_{ad2}.

For the LI 2H-$MoTe_2$, the lowest accuracies were found with V_{Mo}. In left and middle panels of Figure S15**b**, the blue arrow denotes the mis-classified to Perfect; the ground truth of V_{Mo}. Obviously, as indicated by white circles, there exist a Mo_{int} in a blue unit cell (ground truth) *i.e.*, there exist couple of V_{Mo} and Mo_{int}, in a unit cell. The FCN model essentially confuses the defect types since we targeted to define the one-type-of defect in a unit cell. The pair of V_{Mo}-Mo_{int} defects in a unit cell was also confirmed in Figure 3b, which was revealed by 532-nm laser-illumination. As mentioned in the conclusion section, the interstitial defects are challenging due to the random-spatial distribution in a crystal matrix. Also, the absolute number of V_{Mo} was very small, since V_{Mo} is less likely to be transpired than V_{Te1} as mentioned in results section. These two types of low accuracies for Te_{ad2} (V_{Mo}) for 2H-VA 2H-$MoTe_2$ (LI 2H-$MoTe_2$) can be improved by compiling the imaging data and database. More dependable analytic results would be achieved and expanded to more types of point defect types.

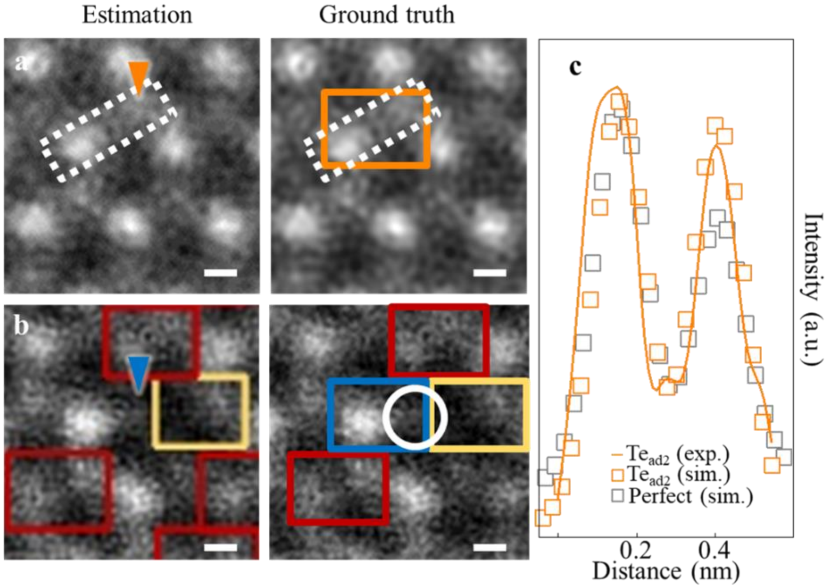

Figure S15. a-b (Left) and (right) Estimated point defects and ground truth of VA 2H-$MoTe_2$ and LI 2H-$MoTe_2$, respectively. Scale bars; 0.1 nm. The color-codes are the same as Figures 2-3. Note that the orange (blue) arrow indicates the mis-classified to Perfect; while the ground truth of Te_{ad2} (V_{Mo}) for VA 2H-$MoTe_2$ (LI 2H-$MoTe_2$). The white circle denotes the Mo_{int}. **c** Intensity profile extracted from white dotted diagonal rectangles in **a**. Orange solid line; experimental intensity profile of Te_{ad2}, Open orange (gray) squares; simulated intensity profile of Te_{ad2} (Perfect).

Supporting References

1. Liu, X.; Qu, D.; Yuan, Y.; Sun, J.; Yoo, W. J. Self-Terminated Surface Monolayer Oxidation Induced Robust Degenerate Doping in $MoTe_2$ for Low Contact Resistance. *ACS Appl. Mater. Interfaces* **2020**, *12*, 26586–26592.

2. Cho, Y.; Park, J. H.; Kim, M.; Jeong, Y.; Ahn, J.; Kim, T.; Choi, H.; Yi, Y.; Im, S. Fully Transparent p-$MoTe_2$ 2D Transistors Using Ultrathin MoO_x/Pt Contact Media for Indium-Tin-Oxide Source/Drain. *Adv. Funct. Mater.* **2018**, *28*, 1801204.

3. Liang, Q.; Gou, J.; Zhang, Q.; Zhang, W.; Wee, A. T. S. Oxygen-induced controllable p-type doping in 2D semiconductor transition metal dichalcogenides. *Nano Res.* **2020**, *13*, 3439–3444.
